# Human Papillomavirus Infection in Head and Neck Squamous Cell Carcinomas: Transcriptional Triggers and Changed Disease Patterns

**DOI:** 10.3389/fcimb.2020.537650

**Published:** 2020-12-02

**Authors:** Nikita Aggarwal, Joni Yadav, Kulbhushan Thakur, Rakhi Bibban, Arun Chhokar, Tanya Tripathi, Anjali Bhat, Tejveer Singh, Mohit Jadli, Ujala Singh, Manoj K. Kashyap, Alok C. Bharti

**Affiliations:** ^1^Molecular Oncology Laboratory, Department of Zoology, University of Delhi, Delhi, India; ^2^Amity Medical School, Stem Cell Institute, Amity University Haryana, Amity Education Valley Panchgaon, Gurugram, India

**Keywords:** oral cavity, transcription factor, oropharynx, prognosis, molecular markers, squamous cell carcinoma, human papillomavirus

## Abstract

Head and neck squamous cell carcinoma (HNSCC) is a heterogeneous group of cancers. Collectively, HNSCC ranks sixth in incidence rate worldwide. Apart from classical risk factors like tobacco and alcohol, infection of human papillomavirus (HPV) is emerging as a discrete risk factor for HNSCC. HPV-positive HNSCC represent a distinct group of diseases that differ in their clinical presentation. These lesions are well-differentiated, occur at an early age, and have better prognosis. Epidemiological studies have demonstrated a specific increase in the proportions of the HPV-positive HNSCC. HPV-positive and HPV-negative HNSCC lesions display different disease progression and clinical response. For tumorigenic-transformation, HPV essentially requires a permissive cellular environment and host cell factors for induction of viral transcription. As the spectrum of host factors is independent of HPV infection at the time of viral entry, presumably entry of HPV only selects host cells that are permissive to establishment of HPV infection. Growing evidence suggest that HPV plays a more active role in a subset of HNSCC, where they are transcriptionally-active. A variety of factors provide a favorable environment for HPV to become transcriptionally-active. The most notable are the set of transcription factors that have direct binding sites on the viral genome. As HPV does not have its own transcription machinery, it is fully dependent on host transcription factors to complete the life cycle. Here, we review and evaluate the current evidence on level of a subset of host transcription factors that influence viral genome, directly or indirectly, in HNSCC. Since many of these transcription factors can independently promote carcinogenesis, the composition of HPV permissive transcription factors in a tumor can serve as a surrogate marker of a separate molecularly-distinct class of HNSCC lesions including those cases, where HPV could not get a chance to infect but may manifest better prognosis.

## Introduction

Squamous cell carcinoma (SCC) of head and neck (H&N) region is a set of highly heterogeneous group of malignancies that collectively pose a major health challenge worldwide. Last 50 years have witnessed a significant shift in the incidence and prevalence of SCC of different constituent subsites. These changes have been linked to changes in exposure to various known carcinogens and risk factors. Traditionally, head and neck squamous cell carcinoma (HNSCC) have been linked to tobacco and alcohol abuse; however, cancer registry data and several cohort studies have documented existence of infection of human papillomavirus (HPV) that have been abnormally detected in oral cavity and tumor tissues of H&N region (Syrjanen et al., 1983; Syrjanen et al., 1987; Syrjanen et al., 1992; Franceschi et al., 1996; Sankaranarayanan et al., 1998; Herrero, 2003; Herrero et al., 2003; Syrjanen et al., 2017). Large clinico-epidemiological studies have revealed that a subset of H&N tumors are specifically linked to HPV infection, which otherwise is traditionally associated with causation of SCC of the uterine cervix and other ano-genital organs (Berman and Schiller, 2017).

A deeper understanding of HPV-positive HNSCC tumors and their response to traditional chemo-radiotherapy revealed existence of a distinct disease type that do not associate with traditional risk factors like tobacco and alcohol abuse (Smith et al., 2004b; Chaturvedi et al., 2015). These are newly emerging tumors and showed increasing incidence. Due to strong association of sexual transmission of HPV in uterine cervix, HPV-positive tumors are associated with sexual behaviors in vastly changing societal practices; however, other modes by which HPV enters in oral cavity also contribute substantially.

Studies performed in initial two decades demonstrated that the affected individuals with HPV-positive HNSCC were of younger age group and showed typically well-differentiated tumors (Pintos et al., 1999; Gillison et al., 2000; Hammarstedt et al., 2006). These prominent clinico-epidemiological differences paved the way for detailed molecular studies to dissect differences in HPV-positive and HPV-negative HNSCC. Later studies subsequently pointed to salient differences at gene expression profiles among these two types of tumors (Cancer Genome Atlas, 2015). Mechanistic studies revealed that these differences can be traced back to the expression and activity of the host cell transcription factors, which work as molecular switches and control cellular behavior. Some of these transcription factors also control establishment of early phase of HPV infection in host cells (Mishra et al., 2006; Gaykalova et al., 2015; Verma et al., 2017). Present review is aimed to compile the available evidence that highlights the involvement and role of key transcription factors in HPV-positive and HPV-negative HNSCC that influence the pathological manifestation of the disease and thus may be useful in the prognostication and effective clinical management of H&N cancers.

## HPV in H&N Region and Association With the Cancer Causation

**Figure 1:**
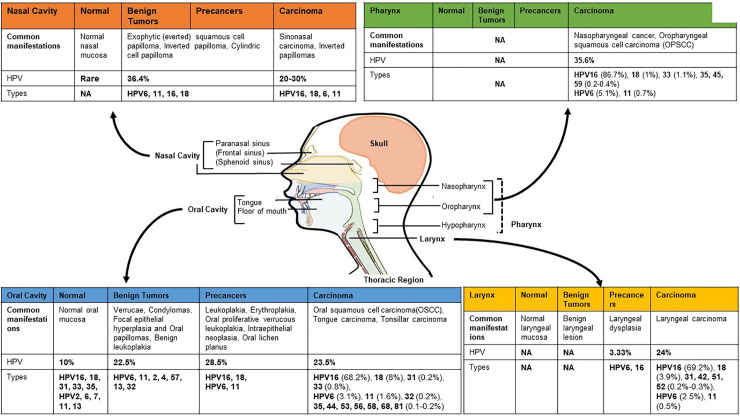
HPV footprint in different anatomical sub-site of head and neck region. Site-wise HPV prevalence and type distribution in different regions of head and neck broadly classified as oral cavity (anterior and middle tongue, floor of the mouth, gingiva, gum, palate, lip mucosa and other sites of the mouth), pharynx (oropharynx with tonsils and base of tongue, nasopharynx, and hypopharynx), larynx and nasal cavity with paranasal sinus (frontal sinus, sphenoid sinus). Hypopharynx, which show low, or no HPV positivity, is sometimes included with laryngeal region for HPV prevalence studies. Data presented in the figure is derived from different systematic reviews and meta-analyses (Miller and Johnstone, 2001; Syrjanen, 2003; Kreimer et al., 2005; Sarkola et al., 2008; Rautava and Syrjanen, 2011; Syrjanen et al., 2017; Onerci Celebi et al., 2018). [Image originally produced by Macmillan Cancer Support and reused with permission].

With the exception of a small fraction of tumors of salivary gland, SCC collectively constitutes more than 90% of all cancers of the H&N region. HNSCC originate from squamous cells of different organ sites below the skull base and above the thoracic inlet. Because of the heterogeneity, HNSCC have been designated with a specific identifier under International Classification of Diseases-10 (ICD10) for individual anatomical site of origin of the tumor. Broadly, H&N region can be divided into oral cavity, nasal cavity, pharynx and larynx (**Figure 1**). Cancers of oral cavity comprise tongue, floor of the mouth, gingiva, gum, palate, lip mucosa and other sites of the mouth. Cancers of pharyngeal region include nasopharynx, oropharynx (base of tongue, palatine tonsils, soft palate, lingual tonsils and posterior and lateral oropharyngeal walls), and hypopharynx. In addition, cancers of larynx, paranasal sinuses, and nasal cavity are also included in HNSCC (Institute, 2017). Collectively, the total burden of cancers in H&N region (ICD-10: C00-13, and 32) sum up to 2,287,731 cases with an annual incidence and mortality rate of 887,659 and 453,307, respectively (Bray et al., 2018). These cancers are overrepresented in men (3:1) and are classically tobacco-associated. There is a significant variation in the incidence of cancers by subsites (Shield et al., 2017). The variation have been attributed to contribution of additional factors such as alcohol consumption, chewing of betel quid with or without tobacco, smoking of local cigars (Sankaranarayanan et al., 1998), environmental exposure to organic solvents, wood and steel dust (Shangina et al., 2006), poor oral hygiene (Rosenquist, 2005), dietary habits, genetic susceptibility (Louvanto et al., 2018), and exposure to infections of Epstein-Barr virus (Young et al., 1988), and of HPV (Herrero, 2003; Herrero et al., 2003). Among others, infection with HPV has emerged as an independent risk factor for a subset of H&N cancers. With changing lifestyle, HPV positive cancers are increasing at an alarming rate (Soto et al., 2000; Shah et al., 2017). Several excellent meta-analysis and reviews on HPV prevalence in H&N show significant variations in HPV positivity that range from 0–100% in different studies (Kreimer et al., 2005; Syrjanen et al., 2017). In a comprehensive analysis of 60 studies from different geographical regions, an overall HPV prevalence of 25.9% in HNSCC was reported (Kreimer et al., 2005). Among these, oropharyngeal cancers displayed the highest and increasing HPV positivity over time (Castellsague et al., 2016). Within oropharyngeal tumors, a comparatively higher HPV positivity is reported in tonsillar crypts and base of tongue (Haeggblom et al., 2017).

HPV is a member of family Papillomaviridae that represents more than 205 different types of papillomaviruses (Bernard et al., 2010; Syrjanen, 2018). Apart from the classification on the basis of sequence divergence in HPV *L1* gene into different genera, these double-stranded DNA viruses are also classified based on their epithelial tissue-tropism as mucosal and cutaneous types. Similarly, based on epidemiological correlates i.e. the frequency of occurrence in malignant or benign lesions of uterine cervix, HPV is grouped as high-risk (HPV16, 18, 31, 33, 39, 45, 51, 52, 56, 58, 59, 66, 68, 69, 73, and 82) or low-risk types (HPV6, 11, 40, 42, 43, 44, 54, 61, 70 72, 81, and 89), respectively (Shukla et al., 2009). Among the high-risk HPV, HPV type 16 represents the most prominent HPV infection in both cervical and HNSCC, whereas, the low-risk HPV type 6 and type 11 are associated with benign lesions like papillomas and warts of H&N as well as genital regions. Different anatomical subsites reportedly have a specific spectrum of HPV infection and genotypes that differ with clinical manifestation of the lesion (**Figure 1**). Even though pharynx encompasses hypopharynx, oropharynx, nasopharynx, all the three sites show distinctive variations in susceptibility to HPV infection. Oropharynx has the highest occurrence of high-risk HPV infections. Nasopharynx is attributed more to Epstein-Barr virus than the HPV. The overall prevalence of HPV is higher in oropharyngeal cancer [Odds Ratio (OR): 14.66] than the oral cavity cancer (OR: 4.06) and laryngeal cancer (OR: 3.23) (Shaikh et al., 2015). Most cancers of hypopharynx and larynx are not related to HPV infection. The larynx is usually infected by low-risk HPVs with less than 2% infections leading to malignant transformation (Torrente et al., 2011; Gama et al., 2016). The type-specific contributions of HPV16/18 in H&N region is around 84.9%, and for all major HPV types (HPV6/11/16/18/31/33/45/52/58) collectively it is 89.7% (De Martel et al., 2017). In a site-wise distribution of HPV types in HPV-attributable HNSCC, HPV16 alone accounted for 86.7% oropharyngeal SCCs (OPSCC), 68.2% for oral SCCs and 69.2% for laryngeal SCCs (Kreimer et al., 2005).

Even though a number of studies have addressed issues related with HPV positivity in H&N region, unlike cervical neoplasia, an accurate estimate of HPV prevalence in H&N remain uncertain due to cumulative effects of a multitude of factors, such as: (i) the techniques that varied greatly in their sensitivity, (ii) all sites in H&N region are not equally susceptible to HPV infection and may not show productive HPV infection, and (iii) the differential risk of opportunistic exposure to HPV infection due to (a) variations in high-risk sexual behavior, or (b) vertical transmission for HPV-positive mothers leading to oro-genital transmission, that significantly differed among different populations.

## HPV-Positive HNSCC as a Molecularly Distinct Subtype

Following detection of HPV antigens in H&N region (Syrjanen et al., 1983; Wookey et al., 2019), efforts in first 20 years were primarily directed towards detecting and enumerating HPV positivity and type-specific distribution in different H&N subsites (Syrjanen et al., 2017). These studies paved the way for large clinico-epidemiological surveys and meta-analyses directed to investigate various risk factors, natural history and thus clinically characterized the HNSCC with reference to their HPV status. These HPV-positive tumors were reported in early stage (Pintos et al., 1999; Smith et al., 2004b; Hammarstedt et al., 2006), well-differentiated histology (Pintos et al., 1999; Gupta et al., 2015), basaloid morphology (Gillison et al., 2000), larger tumors (Gletsou et al., 2018), and either no lymph node involvement (Pintos et al., 1999) or with cystic cervical lymph node positivity (Goldenberg et al., 2008). These tumors showed low risk of second primary malignant neoplasm (Adjei Boakye et al., 2018) with a better overall and disease-free survival (Ragin and Taioli, 2007; Fakhry et al., 2008; Ang et al., 2010; Rischin et al., 2010; Posner et al., 2011; Fakhry et al., 2014). Irrespective of the tissue subtype involved, HPV positivity in HNSCC emerged as strong biomarker associated with better prognosis (Gillison et al., 2000; Wookey et al., 2019).

**Figure 2:**
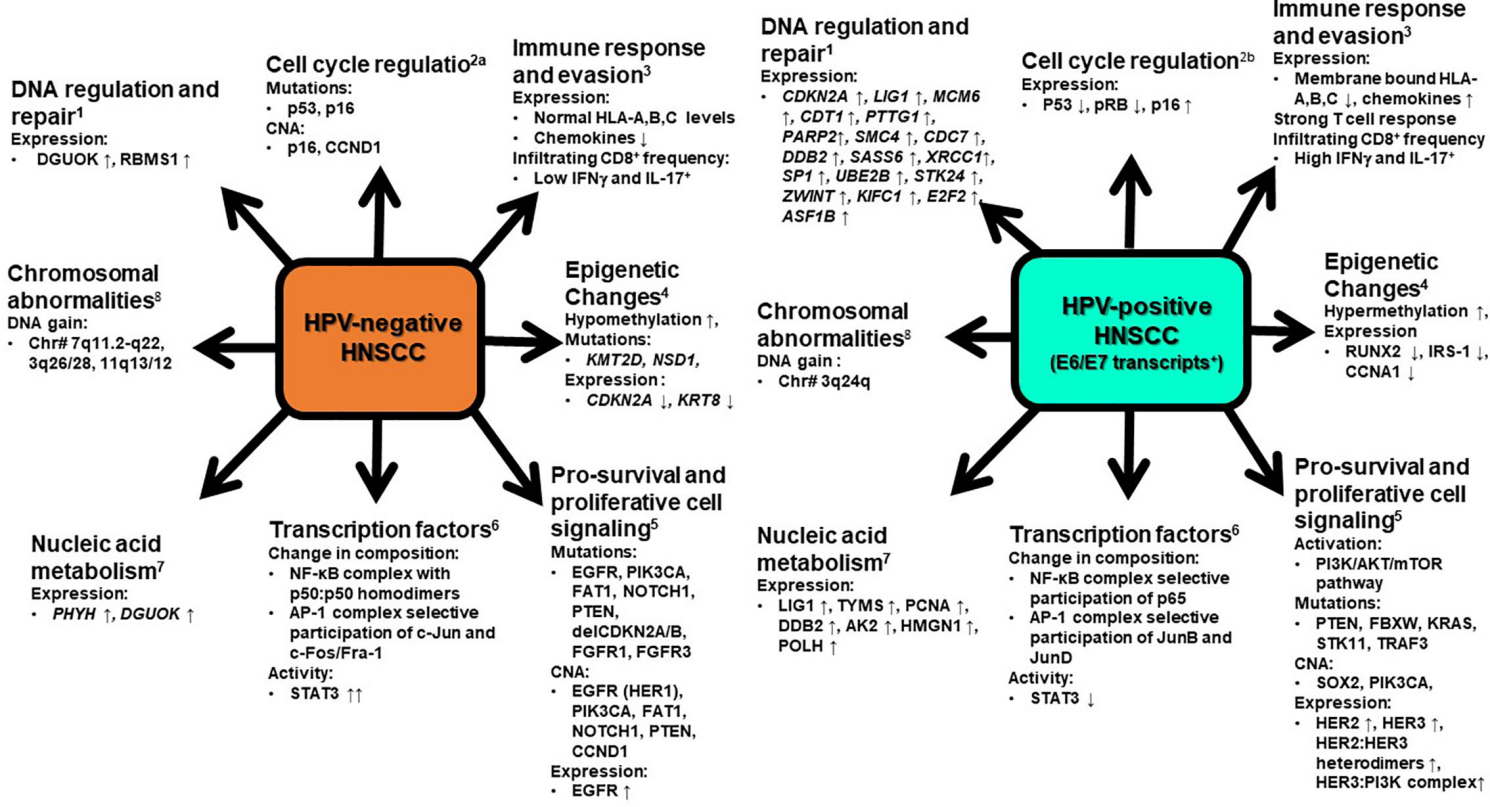
A snapshot of molecular signatures that distinguish HPV-positive and HPV-negative HNSCC. HPV-positive HNSCC that display specified signatures typically possess transcriptionally-active HPV infections. The changes depicted are characteristically displayed by clinically advanced HNSCC. At an early stage or at the time of viral entry all these changes are not evident in potentially malignant cells. CNA, copy number alterations, ↑ - increase, ↓ -decrease. 1. Upregulation of genes involves in DNA regulation and repair (Lohavanichbutr et al., 2009). 2. Cell cycle regulation (Van Houten et al., 2001; Hafkamp et al., 2003). 3. Varied immune response by HPV positive and HPV negative HNSCC (Nasman et al., 2013; Partlova et al., 2015). 4. Epigenetic changes (Sartor et al., 2011; Lindsay et al., 2017; Leemans et al., 2018) 5. Pro-survival and proliferative cell signaling (Chung et al., 2015; Pollock et al., 2015; Seiwert et al., 2015) 6. Modification in expression and activation of different transcription factors (Mishra et al., 2006; Gaykalova et al., 2015; Verma et al., 2017; Gupta et al., 2018). 7. Upregulation of genes involved in nucleic acid metabolism (Lohavanichbutr et al., 2009). 8. Gain in DNA of chromosome (Dahlgren et al., 2003). At an early stage or at the time of viral entry all these changes are not evident in potentially malignant cells.

Comprehensive molecular-genetic studies carried out on primary and metastatic tumors, and cell lines from HNSCC revealed a series of molecular differences among HPV-positive and HPV-negative tumors and the amount of molecular data is currently overwhelming (Leemans et al., 2018). As outlined in **Figure 2**, the differences are manifested through change in gene expression profile or appearance of somatic mutations in gene involved in cell survival and apoptosis, cell cycle, DNA replication, recombination and repair, cellular assembly and localization, nucleic acid metabolism, immune response, signal transduction, and transcriptional and post-transcriptional regulation through action of viral oncogenes or epigenetic silencing, which dominated in HPV-positive tumors (Slebos et al., 2006; Martinez et al., 2007; Lohavanichbutr et al., 2009). On the other hand, mutations either inactivating in tumor suppressor genes or gain of function in oncogenes predominated in HPV-negative HNSCC (Stransky et al., 2011; Lechner et al., 2013; Zhang P. et al., 2014). Since OPSCC sample prevailed in majority of the studies, the HPV-specific signatures can be directly associated with HPV-positive OPSCC.

### Changes in Cell Cycle Regulators

Maiden molecular differences between HPV-positive and HPV-negative HNSCC emerged from studies on p53, pRB and p16 that served as direct cellular targets of viral oncoproteins, E6 and E7 of high-risk HPVs (Van Houten et al., 2001; Hafkamp et al., 2003). HPV-positive tumors characteristically showed a wild type, functionally active p53 gene, whereas HPV-negative tumors invariably harbored inactivating p53 mutations. In addition, p53 mutations were also detected in HPV-positive tumors where the HPV was not transcriptionally-active. Preventing HPVE6 and E7 expression by experimental means restored functional p53 and pRb suppressor pathways in HPV-positive HNSCC (Rampias et al., 2009). E7-mediated pRb is associated with concomitant overexpression of cell cycle inhibitor p16 in HPV-positive tumors (Hafkamp et al., 2003). p16 showed a prognostic value in clinical follow up studies (Licitra et al., 2006; Chung et al., 2015), and it was subsequently referred as surrogate marker of active HPV infection. However, later comprehensive biomarker studies using p16 concordance with active HPV infection showed a false positivity rate of about 20% (Castellsague et al., 2016). Microarrary-based study consistently revealed a higher p16 expression in HPV-positive as compared to normal tissues, but p16 level remained indistinguishable from the normal counterpart in most of the HPV-negative tumors and the difference was not significant. The profile of overexpressed and downregulated genes in these categories significantly differed with a very few overlaps, which is indicative of entirely different tracks of cancer progression events in two HNSCC types (Slebos et al., 2006; Martinez et al., 2007; Lohavanichbutr et al., 2009). Reduced *p53* transcript in HPV-positive tumors was associated with activation of oncogenic pathway genes such as *CDKN2A/CCND1* and other candidate transcript genes such as *SFRP1, CRCT1, DLG2, SYCP2*, and *CRNN* with *SYCP2*, which contribute to genetic instability during development of HNSCC (Masterson et al., 2015).

### Cytogenetic Alterations

Comparative genomic hybridization showed gross chromosomal abnormalities in 3q and 7q region (Dahlgren et al., 2003). HPV-positive tonsillar tumors displayed DNA gain of 3q (72%) with absence of any change in 7q loci, whereas HPV-negative tumors showed a specific gain of 3q (40%) and 7q (40%) loci in sizable number of cases. DNA gain in 3q24 region was associated with increased expression of corresponding genes in the locus (Slebos et al., 2006). Worth noting, the long arm of chromosome 3 (3q26) harbors the *SOX2* and *PIK3CA* genes, which were implicated specifically in HPV-positive tumors (Yarbrough et al., 2007; Lechner et al., 2013).

### Pro-Survival and Proliferative Cell Signaling Pathways

Mutations in genes such as PIK3CA, PTEN, DDX3X, FGFR2/3 and BRCA1/2, TRAF3/CYLD genes, and enriched copy number variations in PIK3CA, KRAS, MLL2/3, NOTCH1, and DNA repair genes were also reported in HPV-positive HNSCC (Chung et al., 2015; Seiwert et al., 2015; Hajek et al., 2017). Likewise HPV-negative HNSCC showed mutations in *TP53, CDKN2A, PIK3CA, CUL3, NSD1*, and* NOTCH* genes. Through a novel proximity based study using VeraTag assay, it was determined that HPV-positive HNSCC exhibited a significant elevated expression of total HER2, total HER3, HER2:HER3 heterodimers, and the HER3:PI3K complex. In contrast, HPV-negative HNSCC exhibited elevated expression of total EGFR(HER1), which contributes to the resistance to EGFR inhibitors in these tumors (Pollock et al., 2015). A cross talk occurs between PI3K signaling, HER3 receptor, and E6 and E7 expression. By RTK profiling, PI3K inhibition led to elevated expression of HER3 receptor, which in turn increased the abundance of E6 and E7 oncogenes to promote PI3K inhibitor resistance (Brand et al., 2018). HNSCC patients with wild type PIK3CA had higher 3-year disease free survival than PIK3CA mutant patients (Beaty et al., 2019).

### DNA Replication and Repair

Differences in components of DNA replication and repair immensely contribute to higher sensitivity to radiation and chemotherapeutic agents displayed by the HPV-positive HNSCC (Liu C. et al., 2018). Owing to the existence of functional p53, strong double strand breaks in DNA override E6-mediated inhibition in p53, leading to cell cycle arrest and induction of apoptosis (Kimple et al., 2013); however, HPV-negative tumors sustain greater damage to DNA before entering the mitotic catastrophe. HPV-positive cells also showed impaired DNA repair (Rieckmann et al., 2013). Overexpressed p16 in HPV-positive HNSCC was found to block homologous recombination-mediated DNA repair by preventing RAD51 (Dok et al., 2014).

### Immune Response

Reduced MHC class I antigens are the hallmark of HPV-positive HNSCC lesions (Nasman et al., 2013). E7 plays a key role in this process and increases susceptibility of HPV-positive HNSCC to natural killer cells (Georgopoulos et al., 2000). HPV-positive HNSCC exhibited enhanced and distinct pattern of immune activation to HPV-positive HNSCC, which included upregulation of genes associated with immune-associated processes, high infiltration rate of CD8^+^IFNγ^+^ T lymphocytes, Tc17 lymphocytes, naïve CD4^+^T lymphocytes, and myeloid DCs along with high level of production of chemokines CXCL9, CXCL10, CXCL12, CXCL17, and CXCL21 (Partlova et al., 2015; Chen et al., 2018). These lesions reported higher ratio of M1/M2 macrophages, lower expression of COX-2 mRNA, and higher expression of PD1 mRNA (Chen et al., 2018).

### Epigenetic Modifications

HPV-negative HNSCC genomes, in general, are hypomethylated and showed higher loss-of-heterozygosity (LOH) and genomic instability as compared to HPV-positive tumors (Richards et al., 2009). HPV-positive tumors showed higher promoter methylation of polycomb repressive complex 2 target genes and increased expression of DNMT3A, whereas higher methylation and lower expression of RUNX2, IRS-1, and CCNA1 was noted in HPV-negative tumors (Sartor et al., 2011). These differences in methylation patterns with larger panels later were found to have prognostic value (Ren et al., 2018).

### Transcriptional Regulation

Pioneering studies conducted by our group showed differential expression and activity of transcription factors in HNSCC with respect to the HPV status (Mishra et al., 2006; Gupta et al., 2015; Verma et al., 2017; Gupta et al., 2018). HNSCC in general showed constitutively active nuclear factor-κB (NF-κB); however, the active complex differed in the composition. HPV-negative tumors had p50:p50 homodimers, whereas p65 participation was detected in HPV-positive tumors (Mishra et al., 2006; Gupta et al., 2018). Similarly, constitutively active activator protein-1 (AP-1) differed in its composition. AP-1 with JunB and JunD participated with c-Fos & Fra-2 in HPV-positive tumors, whereas, c-Jun was the major binding partners forming the functional AP-1 in HPV-negative tumor (Gupta et al., 2015). Subtle changes in composition of these transcription factors have significant impact on the regulation of set of downstream genes (Gaykalova et al., 2015). In contrast to NF-κB and AP-1, signal transducer and activator of transcription-3 (STAT3) was negatively regulated with HPV positivity in HNSCC. These differences were reported in both retrospective and prospectively collected samples by simple IHC procedures (Verma et al., 2017). Analysis of Cancer Atlas data demonstrated defects in TRAF3 and CYLD, which correlated with activation of NF-κB (Hajek et al., 2017).

These evidences collectively suggest presence of molecularly distinct gene expression patterns in HPV-positive and HPV-negative HNSCC, which could potentially contribute to their favorable outcome and better prognosis. However, studies also emphasize essential requirement of HPV to be transcriptionally-active to have a prognostic relevance (Braakhuis et al., 2004; Jung et al., 2010; Janecka-Widla et al., 2020). Further, most of the studies that highlighted HPV-negative signatures also identified yet another subgroup of HNSCC that displayed favorable clinical outcome even in the absence of HPV. These findings emphasize need for greater understanding of the transcriptional state of the infected cells and the cellular players that perform driver role in coupling host transcription with the virus.

## Entry and Establishment of HPV Infection in the Host Cell

In an *in vitro* culture system, the transforming potential of HPV in primary oral keratinocyte is well established (Chu, 2012). However, not all individuals positive for oral HPV DNA develop carcinoma, which is suggestive of cellular heterogeneity in H&N region and individualistic variations in cellular environment that may permit or restrict productive HPV infection (Smith et al., 2004a). Succeeding immunological escape, incident HPV can complete its life cycle in H&N tissues and exhibit genome maintenance, vegetative and replicative phases just like in cervix (Raff et al., 2013; Syrjanen, 2018). However, the exact mechanism and target cells in these tissues are poorly defined. Studies show gingival pockets as reservoirs of HPV infection (Hormia et al., 2005). Even though transcriptionally-active HPV infection is detected in different H&N sites, a well-defined HPV susceptible region in these tissues like squamocolumnar junction in cervix, do not exist. Being an epitheliotropic virus, it targets the basal layer cells of the epithelium that becomes accessible due to micro-abrasions. Cells of the basal layer that undergo keratinizing changes provide a suitable cellular environment for progression of viral life cycle (Harden and Munger, 2017). Transcriptionally active HPV, either detected by expression of viral oncoproteins or their transcripts, has been reported in almost all major H&N sites namely oral cavity (Shroyer and Greer, 1991), tonsillar (Snijders et al., 1992), and oropharyngeal region (Gillison et al., 2000) including larynx (Scheurlen et al., 1986).

**Figure 3:**
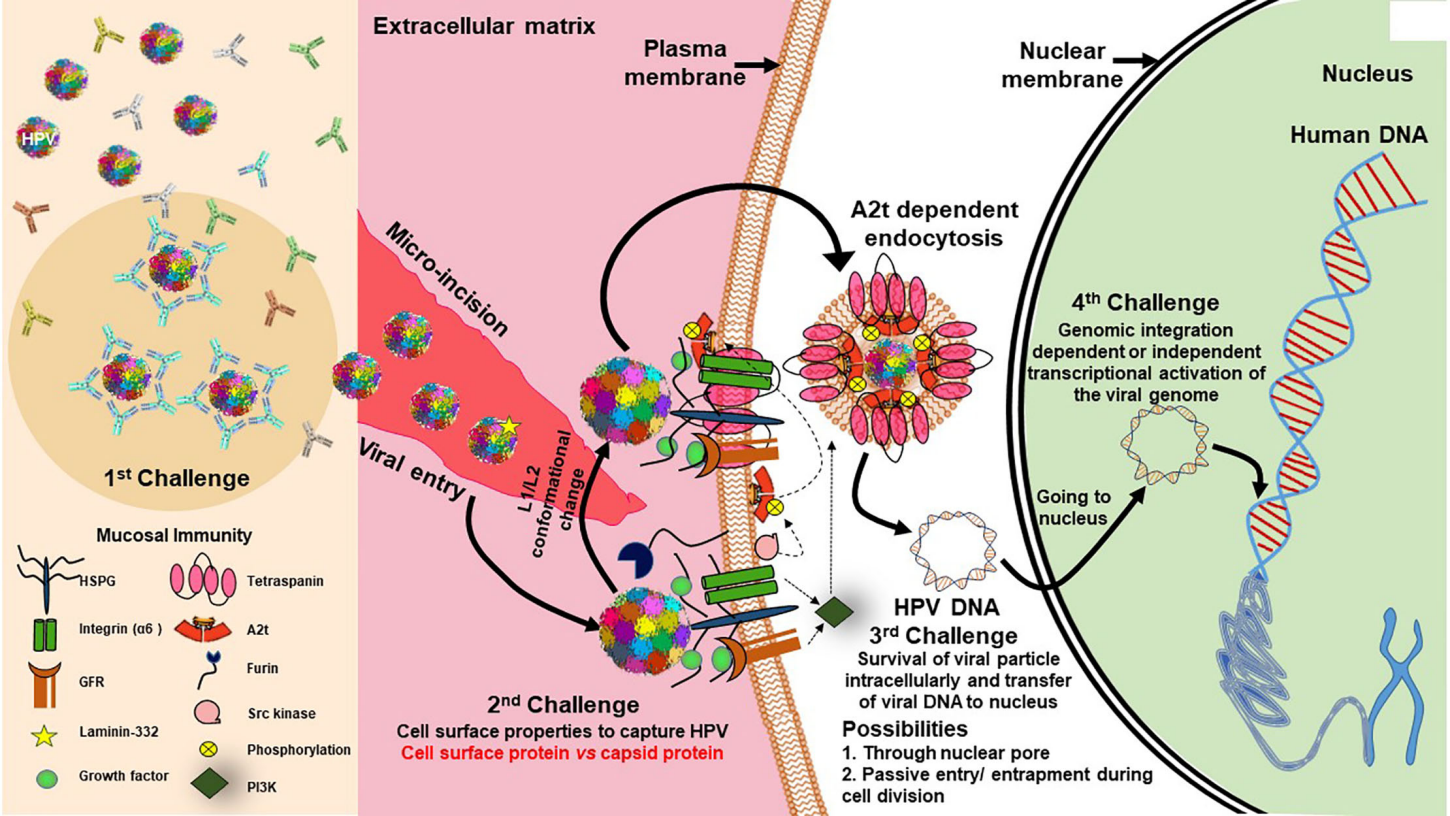
Challenges for establishment of HPV infection in basal epithelial cells of H&N region. Establishment of transcriptionally-active HPV infection in epithelial tissues faces various challenges to establish in the host cell. First challenge is posed by the mucosal immunity against HPV coat proteins that results in clearance by HPV specific antibodies (Jenson et al., 1991). Second challenge is viral entry which is specifically facilitated through micro-incisions and interaction of HPV-HSPG (heparan sulfate proteoglycan)-growth factor complexes with growth factor receptors (Surviladze et al., 2012) that leads to rapid activation of signaling pathways, such as PI3K/Akt/mTOR, which further inhibit autophagy of cell for viral benefit (Surviladze et al., 2013). Further, src kinase phosphorylates annexin A2(AnxA2) at Tyr23 (A2t), HPV recognizes A2t and binds to it in Ca^2+^ dependent manner and A2t dependent endocytosis of HPV particle and support trafficking of virus in cell (Dziduszko and Ozbun, 2013). Third challenge is presented for the viral DNA for survival in intracellular milieu and its transport to the nucleus, which could likely be through a passive entry/entrapment during cell division. Last and most pivotal challenge is presented for its genomic integration and transcriptional activation of viral genome to produce functional E6/E7 transcripts. In the absence of transcriptionally activation of E6 and E7, the lesion is similar to HPV negative lesion. On the other forced expression of E6/E7 can independently derive keratinocyte transformation.

HPV infection, if it has to be tumorigenic, has to overcome several barriers and challenges (**Figure 3**). Further, the cell division and differentiation program of infected host squamous epithelium controls the HPV life cycle (Harden and Munger, 2017). During establishment of HPV infection, the most critical contribution comes from the availability of a set of transcription factors that control expression of early HPV genes. In the absence of these host transcription factors, the virus is defunct and eliminated or just maintains its genome with host cell cycle till the required factors are expressed/activated during the cell differentiation stage or local inflammation. Chronic inflammation serves as a driving force in speeding up carcinogenesis in H&N region (Liu et al., 2015). HPV Infections incapable of undergoing transcriptional activation are non-productive and are either eliminated/cleared from the tissue or do not influence tumorigenic properties of the host cells (Boscolo-Rizzo et al., 2016). Therefore, for tumorigenic activation, HPV genome is entirely dependent on host factors (Kyo et al., 1997). Studies carried out by our group and others in last 5 years have clearly demonstrated the differential expression and activity of host transcription factors particularly AP1, NF-κB and STAT3 in HPV-positive HNSCC or the transcript profile of their downstream genes (Mishra et al., 2006; Gaykalova et al., 2015; Verma et al., 2017). Incidentally, the level of expression and activity of these transcription factors have independent oncogenic and predictive valve due to their more pragmatic role in regulation of variety of cellular functions, particularly in the regulation of inflammation (Iliopoulos et al., 2009). Therefore, HPV-related transcription factors provide a coupling action between inflammation, activation of HPV oncogenes, and development of cancer.

### Transcriptionally-Active HPV Infections in HNSCC: Role of Long Control Region (LCR)

**Figure 4:**
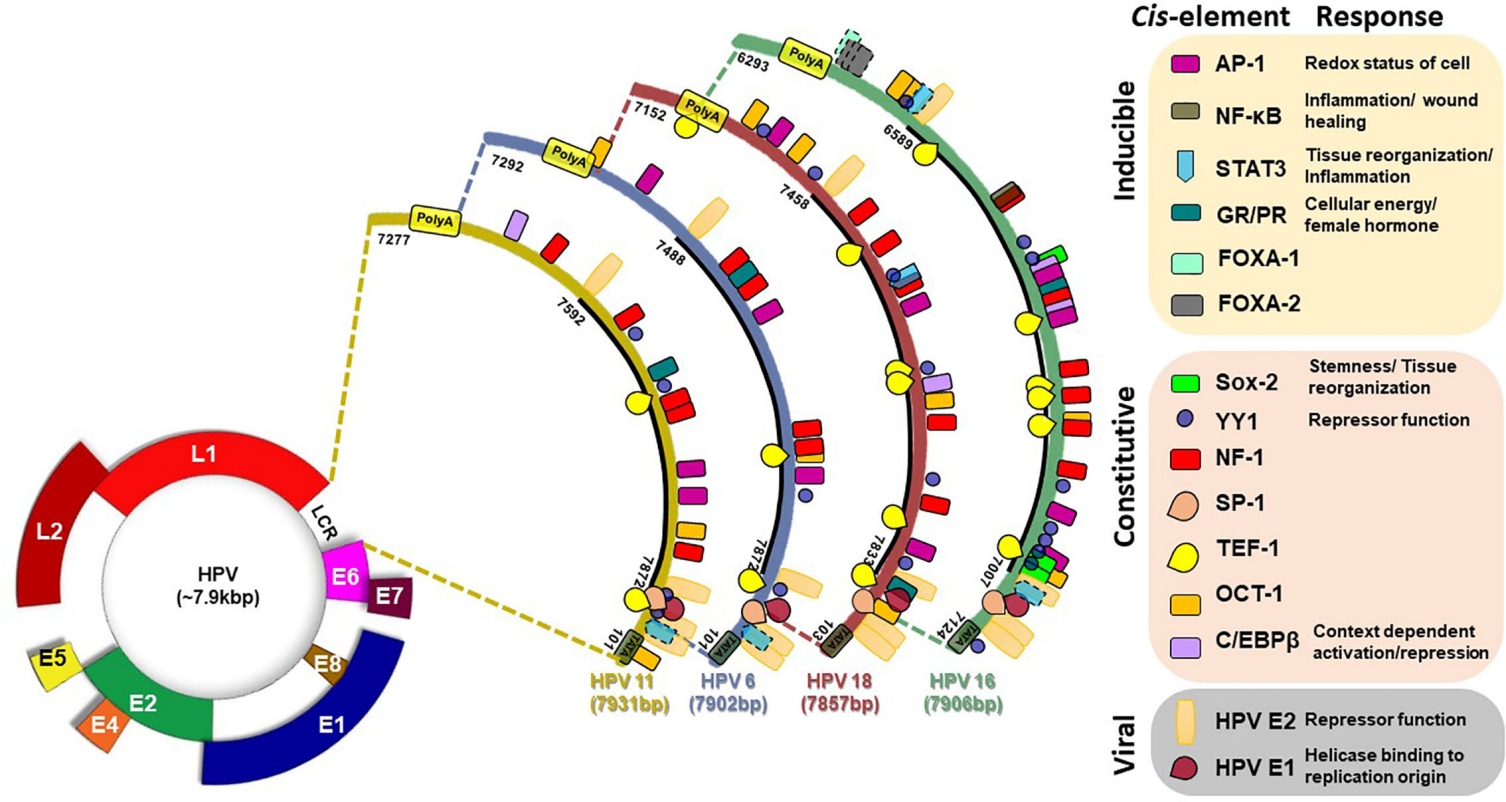
Transcription factor binding site on LCR of HPV16 (831 bp), HPV18 (835), HPV6 (811 bp), and HPV11 (755 bp). Various consensus-binding sites for different transcription factors present on HPV LCR are represented as boxes. Solid line around the box indicate experimentally-validated site, broken line indicate putative site. Consensus-binding sites of AP-1 (Gloss et al., 1989; Chan et al., 1990; Chong et al., 1990; Nakshatri et al., 1990; Thierry et al., 1992; O’connor et al., 1995; O’connor et al., 1996; Wang et al., 2011), STAT3 (Arany et al., 2002), NF-κB (Fontaine et al., 2000), SOX-2 (Martinez-Ramirez et al., 2017), YY1 (O’connor et al., 1995; O’connor et al., 1996), NF-1 (Gloss et al., 1989; Chong et al., 1990; Nakshatri et al., 1990; O’connor et al., 1995), SP-1 (Gloss and Bernard, 1990), TEF1 (Ishiji et al., 1992; O’connor et al., 1995), Oct-1 (O’connor and Bernard, 1995; O’connor et al., 1995), FOXA1 (Sichero et al., 2012; Xi et al., 2017), on HPV16 (NC_001526.4), HPV18 (X05015.1), HPV6 (NC_001355.1), and HPV11 (M14119.1) have been reported in different studies and have been shown as per revised reference sequences in NCBI Nucleotide database.

A ~850bp non-coding region present on viral DNA, designated as Long Control Region (LCR) or synonymously known as Upstream Regulatory Region, constitute over 10% of HPV genome. LCR contains most of the tissue/cell-specific enhancer region and is populated by (i) *cis*-responsive elements that regulate HPV life cycle, (ii) the replication origin on 3’ end where HPVE1 binds, and (iii) the early promoter (p97 in HPV16) that control the expression of oncogenes E6 and E7 (**Figure 4**) (Cripe et al., 1987; Gloss et al., 1987). LCR is the most variable region of HPV genome and regulates transcription and replication of the viral DNA (Pande et al., 2008; Xi et al., 2017; Ribeiro et al., 2018). The LCR of all clinically-relevant genital HPVs are organized similarly (O’connor et al., 1995). Viral oncogenes E6 and E7 produced as a fusion transcript are produced by a promoter which is directly regulated by the LCR. HPV16 LCR, which can be considered as a model for the HPVs, can be subdivided by two E2 binding sites, which serve as landmarks, into three functionally-distinct segments, named as 5’ or distal, the central, and 3’ proximal segment due to their positioning with respect to the early promoter (O’connor et al., 1995).

As depicted in **Figure 4**, typical HPV16 LCR contains binding sites for various host transcription factors like AP-1, NF-κB, STAT3, Sox-2, Glucocorticoid Response Element (GRE)/Progesterone Response Element (PRE), YY-1, NF1 (CAAT binding Transcription Factor 1), SP1, TEF-1, Oct-1 (NFA), cEBP/β, FOXA1, FOXA2, KRF-1, GATA3, CDP, SOX9, TEF-2, and PHOX2A, which were identified by different bioinformatics and experimental approaches (Gloss et al., 1987; Gloss et al., 1989; Chan et al., 1990; Gloss and Bernard, 1990; Chong et al., 1991; Bauknecht et al., 1992; Ishiji et al., 1992; Thierry et al., 1992; O’connor and Bernard, 1995; Fontaine et al., 2000; Arany et al., 2002; Sichero et al., 2012; Martinez-Ramirez et al., 2017; Xi et al., 2017). Based on the functional role of the corresponding transcription factors, it is predicted that these elements assist the virus to fine-tune its response to the immediate environment such as redox status, local inflammation, cellular reorganization & reprogramming, cellular energy status, and most importantly the hormonal status among others. DNase footprinting, DNA binding and reporter assays helped to establish the functional role of different element detected in the LCR by bioinformatics approaches. Not all sites predicted from bioinformatics (underlined) could be validated in experimental system, whereas some of them either lacked functional activity (lacked DNA binding) (Martinez-Ramirez et al., 2017), or were inconsequential to the enhancer function of the LCR (Sichero et al., 2012). In addition, a few more transcription factors were found to interact with the LCR (Carson and Khan, 2006) even though their binding sites were not detectable by bioinformatics tools, and expected to be secondary interactions.

Minor changes in sequences within LCR region can lead to loss or addition of the binding sites for transcription factors or the efficiency of their interaction (Dong et al., 1994; Pande et al., 2008; Sichero et al., 2012; Xi et al., 2017). For example, NF-κB binding site in the HPV16 LCR promoter is localized in a small region exhibiting enhancer activity (Chong et al., 1990; Fontaine et al., 2000). However, the binding site of NF-κB contains two mismatches in comparison to the consensus NF-κB Igκ in the HPV16 LCR (Sen and Baltimore, 1986). The NF-κB site also overlaps with a NF-1 binding site. Therefore, it is possible that NF-κB competes/cooperate with NF-1 for DNA binding (Fontaine et al., 2000). Congregation of several sites in a small region also underlines a strong interaction, both positive and negative, among different transcription factors. Host factor YY-1 and viral E2 strikingly have repressor functions (Gloss and Bernard, 1990) and loss of their corresponding sites by mutations or by epigenetic mechanism promote the enhancer function of LCR (Bauknecht et al., 1992; Dong et al., 1994; Reuschenbach et al., 2015). Some of these transcription factors work on palindromic elements. In case of these elements, LCR conserves only half site whereas other half site is highly variable. Therefore, slight change in the non-conserved half site strongly influence transcription factor docking and resultant enhancer function of LCR.

AP-1, a redox sensitive transcription factor (Angel and Karin, 1991), specifically binds to the *cis*-regulatory elements identical or related to the consensus sequence TGAGTCA of HPV LCR. Four AP-1 binding sites on HPV16 LCR, three sites each on HPV18 and HPV6 LCR, and two sites on HPV11 LCR have been described (Gloss et al., 1989; Chan et al., 1990; Chong et al., 1990; Nakshatri et al., 1990; Thierry et al., 1992; O’connor et al., 1995; O’connor et al., 1996; Wang et al., 2011) (**Table 1**). Efficient activation of E6/E7 promoter requires the integrity of all the AP-1 elements of HPV16 LCR and mutation in any one cannot be complemented by other motifs (Nakshatri et al., 1990; Thierry et al., 1992). Similarly, binding of FOXA1 and MYC with the promoter and regulatory sequences of HPV16 and HPV18 has been shown *in vivo*. However, only overexpression of FOXA1 enhanced the LCR activity (Sichero et al., 2012). Two putative GATA3 binding sites within the LCR of HPV18 (7205–7210, TGATTG; 7532–7537 TAATCT) were also reported (Sichero et al., 2012). Three functional binding sites of SOX2 were found in the HPV16-LCR (Martinez-Ramirez et al., 2017). C/EBPβ element showed a context dependent action on HPV LCR. It acted as repressor for HPV11 in keratinocytes as levels of both HPV11 transcripts and HPV DNA increased after treatment with oligomers containing the c/EBPβ DNA binding motif (Wang et al., 1996). c/EBPβ-YY1 complex contributed to cell-type-specific HPV18 LCR activity (Bauknecht et al., 1996). c/EBP binding site overlaps with AP-1 in HPV16 LCR and mutation in this composite site that also eliminates AP-1 binding show increases transcription from early promoter (Liu et al., 2002). Together, these observations reflect a strong influence of host transcription factor composition in the target tissue on expression of viral oncogenes that in turn modify the cellular environment leading to manifestation of pathological characteristics of the HPV-positive tumors.

**Table 1:** Transcription factor binding site on LCR of HPV16 (831 bp), HPV18 (835), HPV6 (811 bp), and HPV11 (755 bp).

Transcription factor(Consensus*)	HPV16		HPV18		HPV6		HPV11		References
	Sequence	Position	Sequence	Position	Sequence	Position	Sequence	Position	
**AP1** **(TGANTCA)**	**TGAATCA**	6769–6775	**GTGGTATG**	7349–7356	**TGACTCA**	7435–7440	**TGCATGACTAAT**	7732–7743	(Gloss et al., 1989; Chan et al., 1990; Chong et al., 1990; Nakshatri et al., 1990; Thierry et al., 1992; O’connor et al., 1995; O’connor et al., 1996; Wang et al., 2011)
	**TGTGTCA**	6786–6792	**TGGTATTA**	7603–7610	**TGTTTAA**	7518–7524	**TGGATTGCAGCCAA**	7776–7790	
	**TTAGTCA**	6949–6955	**TGACTAA**	7792–7798	**TTGTAGCA**	7781–7788			
	**TTAGTAT**	7103–7109							
**NF-κB** **(GGGRNNYYCC)**	TGCCAAATCCC	6691–6702							(Fontaine et al., 2000)
**STAT3** **(TT(N)_4-6_AA)**	*TTCAACCGAA*	6584–6594	**TTGAACAA**	7561–7568	*TTCAACCGAA*	46–55	*TTCAACCGAA*	46–55	(Present study) (Seidel et al., 1995)
	*TTACAAGCAA*	7015–7024							(Xi et al., 2017)
**SOX-2** **(CAATGG/CATTGTT)**	*CATTGTT*	6736–6742							(Martinez-Ramirez et al., 2017)
	*CATTGTT*	6958–6964							
	*CACATGG*	6979–6985							
**GRE/PRE (GGTACANNNTGTTCT)**	**TGTACATTGTGTCAT**	6779–6793	**AGCACATACTAT ACT**	7839–7853	**GGTACACATTG CCCT**	7630–7644	GGTACATATTGCCCT	7674–7688	(Chan et al., 1989)
**YY1** **(CCGCCATNTT)**	**CCATTTTGTA**	6573–6582	*CCATTTT*	7343–7349	*ACATTTT*	7791–7797	*ACATTTT*	7620–7626	(O’connor et al., 1995; O’connor et al., 1996)
	**CCATTCCATT**	6730–6739	*CCATTTT*	7443–7449	*ACATATT*	7885–7892	*ACATATT*	7677–7683	
	**CCATTGTTTT**	6736–6745	*ACATATT*	7554–7560			*ACATATT*	7914–7920	
	**ACATGAACTG**	6931–6940	*ACATATT*	7692–7698			*ACATCTT*	8–15	
	**TCATACATTG**	6953–6962	*ACATTCT*	7759–7765					
	**ACATTGTTCA**	6957–6966	*ACATAGT*	7808–7814					
	**TCATTTGTAA**	6964–6973							
	**ACATGGGTGT**	6980–6989							
	**GACATTTTATG**	7118–7127							
**NF1** **(TTGGC)**	**TGCCAA**	6691–6696	**TTGGC**	7475–7479	**TTGCC**	7559–7563	**TTGCC**	7526–7530	(Gloss et al., 1989; Chong et al., 1990; Nakshatri et al., 1990; O’connor et al., 1995)
	**TGCCAA**	6724–6729	**CTGGCA**	7513–7518	**TTGCC**	7655–7659	**TTGCC**	7603–7607	
	**CGCCAA**	6811–6818	**TTGGC**	7582–7586	**TTGCC**	7738–7742	**TTGCC**	7682–7687	
	**TTGGCT**	6849–6854	**TTGGC**	7732–7743	**TTGGC**	7776–7780	**TTGCC**	7699–7703	
	**TTGGCA**	6879–6885	**TTGGC**	7776–7790			**TTGGC**	7805–7809	
	**GGCCAA**	6904–6909							
**SP1 (GGGNGG)**	**GGGCGT**	7070–7075	*GGGAGT*	35–40	**AGGAGG**	28–33	**AGGAGG**	28–33	(Tan et al., 1992)
**TEF-1** **(TACATACTTC)**	**TGCATGCTTT**	6601–6610	*TGCATTGTAT*	7209–7218	*CACATTTTT*	7780–7788	*TACATATTGC*	7676–7685	(Ishiji et al., 1992; O’connor et al., 1995)
	**TACATTGTGT**	6781–6790	*TACATATTTT*	7553–7562	*TACATATTTC*	7884–7893	*TACATATTTC*	7903–7922	
	**TACATACCGC**	6824–6832	*CACATATTTT*	7691–7700					
	**CACATATTTT**	6840–6849	*TGCATACTTG*	7704–7713					
	**TGCATATTTG**	6873–6882	*TACATAGTTT*	7807–7816					
	**TACATTGTTC**	6956–6965	*CACATACTAT*	7841–7850					
**OCT-1** **(AATTGCAT)**	***ATTTTGTAG***	6555–6563	AACTGTAT	7333–7340	ATGTGTAT	7385–7392	AAAAGCAT	7795–7802	(O’connor and Bernard, 1995; O’connor et al., 1995)
	***ATTTTGTAG***	6575–6583	AACTGCAC	7403–7410	AAAAGCAT	7766–7773	ATTAGCAG	84–91	
	***AATTGCAT***	6870–6877	AATTGCAT	7721–7728					
	***AACTGCAC***	6974–6981	AATTGTAG	16–23					
**c/EBPβ** **(TKNNGNAAK)**	**ACTACTGAAT**	6763–6773	**CTTAAGTAA**	7713–7722			**TTGTGCAAT**	7451–7459	(Bauknecht et al., 1996; Wang et al., 1996; Liu et al., 2002)
	**ATTGTGTCA**	6784–6792							
**FOXA-1** **(MAWTRTTKRYTY)**	*CACGTG*	6404–6409							(Xi et al., 2017)
**FOXA-2** **(MAWTRTTKRYTY)**	***CACGTG***	6404–6409							(Martinez-Ramirez et al., 2017)
	***CGTGTGTA***	6406–6413							
	***GTGTATGTGTTT***	6409–6420							

*Consensus sequence represented with IUPAC codes to denote degeneracy. NA; Italic—Bioinformatics analysis; Bold—Experimental result; Underline—Putative binding sites.

**Figure 5:**
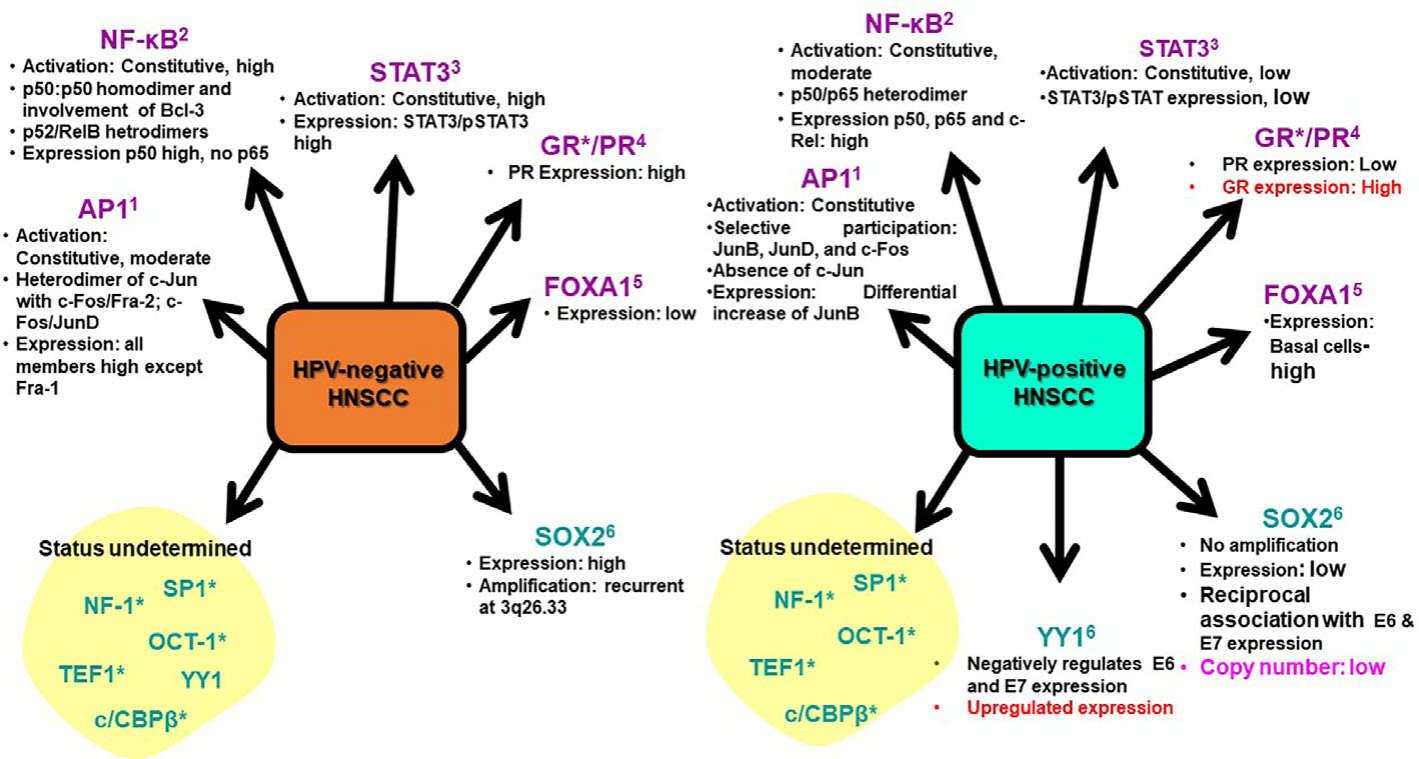
Expression and activity of HPV-related transcription factors in HNSCC. HPV-positive and HPV-negative HNSCC displayed distinguishable transcriptional profiles. Transcription factors broadly classified as inducible (Purple) and constitutively-active (Blue) include: 1. AP1 (Gupta et al., 2015), 2. NF-κB (Fontaine et al., 2000; Bano et al., 2018; Zhang et al., 2018) 3. STAT3 (Gaykalova et al., 2015; Verma et al., 2017), 4. GR/PR (Mohamed et al., 2018; Kost et al., 2019) 5. FOXA1 (Karpathiou et al., 2017; Li et al., 2019) 6. SOX2 (Gut et al., 2018; Dogan et al., 2019) 7. YY1 (Ferris et al., 2005; He et al., 2011). *No specific study in HNSCC. Red text: Data from cervical cancer; Pink text: Data of vulvar carcinoma.

Expression and activation of regulatory host cell factors determine the magnitude, and duration of viral transcription. The transcription factors can be broadly classified as inducible and constitutive based on the fluctuations in their expression and activity levels in the cell. transcription factors AP-1, NF-κB, STAT3, GR, PR, and FOXA1/2 are inducible in nature and their aberrant expression and constitutive activation play an independent role in carcinogenic inflammation, transformation and maintenance of cancer stemness (Angel and Karin, 1991; Rinkenbaugh and Baldwin, 2016). Almost all of these transcription factors have pivotal cellular functions and their temporal expression or changed activation state is sufficient to alter the physiological state of the cell undergoing tumorigenic transformation. Research carried out in recent years have established differential expression and activation of these transcription factors among HPV-positive and negative HNSCC (Gaykalova et al., 2015; Gupta et al., 2015; Verma et al., 2017; Gupta et al., 2018) (**Figure 5**). Therefore, understanding the expression and activity of host transcription factors that could interact or alter the function of LCR in relation to active viral infection, have been an area of active research in HPV biology and development of anticancer therapeutics. Following section is aimed to review the evidence related to the level of expression and role of these transcription factors in HNSCC in general and HPV-driven HNSCC in particular (**Table 2**).

**Table 2:** Expression and activity of key HPV-related transcription factors in normal and tumor tissues of H&N region.

Transcription factor (family members)	Specimen	Transcription factor Activation status	Expression level of transcription factor family members	HPV-specific information	References
**AP-1** [c-Jun (39 kDa), JunB (36 kDa), JunD (35 kDa) c-Fos (41 kDa), FosB (36 kDa), Fra-1 (29 kDa), Fra-2 (35 kDa)]	HNSCC tumors (n = 7) and adjacent control tissues	Increased binding of AP-1 in HIV LTR	Nil	Nil	(Zoumpourlis et al., 1994)
	HNSCC cell lines UM-SCC-1, -9, -11A, -11B, and-38	Active AP-1 with c-Jun, JunB, JunD and Fra-1 as major participants; Loss of AP-1 site reduced IL-8 promoter activity	Nil	Nil	(Ondrey et al., 1999)
	HNSCC cell lines UM-SCC-9 and -11B	IL-1α-induced activation of AP-1; Loss of AP-1 site moderately reduced IL-8 promoter activity	Nil	Nil	(Wolf et al., 2001)
	HPV16 E7 transgenic mouse model with dominant negative c-Jun under human K14 promoter	c-Jun associated AP-1 activation lost; Prevention of chemical-induced skin papillomas	NIL	Increased HPV16 E7 expression in DN-c-Jun mice; more papillomas	(Young et al., 2002)
	HNSCC cell lines UM-SCC-9 and -11B	EGFR promoted AP-1 activity and downstream expression of VEGF	Nil	Nil	(Bancroft et al., 2002)
	HPV immortalized human oral keratinocytes- HOK-16B;	Acetaldehyde activates AP-1 containing c-Jun	Increased *c-jun* mRNA and protein accumulation	HPV-transformed cells	(Timmons et al., 2002)
	HNSCC cell lines: UM-SCC 1, 5, 6, 9,11A, 11B, 22A, 22B, 38, and 46	Heterogeneity in cell lines with respect to AP-1 regulated gene expression	Nil	Nil, (Cell lines differed in their p53 expression	(Yan et al., 2007)
	FFPE-tissues of oral leukoplakias, with different degrees of epithelial dysplasia, and OSCC (n = 50); (HPV positivity-24%)	According to degree of oral dysplasia withing lesion c-Jun nuclear expression increased and greatest expression and nuclear localization in OSCC	Increased expression of c-Jun with increasing severity of the lesion	Malignant progression mediated by c-Jun is independent of the presence of HPV in oral carcinogenesis	(Acay et al., 2008)
	Fresh oral tissue specimens (n = 100)	Increased activation of AP-1 with increasing disease severity	Presence of JunD with c-Fos in AP-1 complex in majority of oral cancer; JunB showed sporadic increase in a subgroup	Nil	(Mishra et al., 2010)
	HPV immortalized human oral keratinocytes- HOK-16B HNSCC cell line- UM-SCC-9, 11A, 11B, 38	Tobacco-carcinogen induced AP-1 reporter gene activity; AP-1 activity dependent on c-Fos expression	c-Fos expression induced by tobacco carcinogens	IL-8 and VEGF expressed both in HPV-transformed oral keratinocytes and HNSCC cell lines	(Swenson et al., 2011)
	Fresh tongue tissue biopsies (n-100), (HPV positivity-28%) Tongue cancer cell lines-HPV16 positive- UPCI:SCC090; HPV negative- AW13516	Increased activation of AP-1 with increasing disease severity; Upregulation of downstream target genes: cyclin D1, c-myc, Bcl-xl, MMP-9, EGFR Composition: c-Jun, c-Fos, Fra-2 in HPV-negative and JunD, JunB, c-Fos and Fra-2 in HPV-positive tumors	Higher expression of all Jun and Fos family members except Fra-1 which showed reciprocal kinetics	Selective participation of JunD and JunB only in HPV16 positive tumors and cell lines; absence of c-Jun in HPV-positive	(Gupta et al., 2015)
	HNSCC cell line: HPV-positive- 93VU-147T	Inhibition of AP-1 activation and change in active AP-1 by curcumin	Curcumin induced loss of AP-1 c-Jun, JunD and JunB in HPV-positive oral cancer cells	Nil	(Mishra et al., 2015)
	HNSCC cell lines – 26; Mouse model of metastasis	Nil	JunB, Fos, Fra-1, JunD overexpressed in cells with metastatic potential	Nil	(Hyakusoku et al., 2016)
	Prospectively collected fresh biopsies - 116 and FFPE -30 from OSCC/OPSCC; Total tissue analyzed (n = 116), FFPE (n = 66)	Nil	Overexpression of JunD, and c-Fos in SCC as compared to normal; Overexpression of JunB only in HPV-positive tissues	Differential increase in JunB in HPV-positive; Level of JunD upregulated but not differentially active only HPV-positive cancers	(Verma et al., 2017)
	Human OSCC samples (n = 123)	Elevated expression of active AP-1 component phospho-c-Jun in resistant tumors; Active AP-1 strongly correlated with bcl-2 overexpression	c-Jun overexpression in chemo-radioresistant tumors	Nil	(Alam et al., 2017)
	HNSCC cell lines: HPV-negative- CAL33^Res^; HPV-positive- UM-SCC-47	Overexpression of c-Jun target gene *AXL*	Silencing of c-JUN and c-FOS expression downregulated AXL expression and enhanced the sensitivity of HPV negative cells	Targeting AP-1 enhanced the antitumor efficacy of BYL719 against HPV positive HNSCC	(Badarni et al., 2019)
**NF-κB** [NF-κB1 (50 kDa), NF-κB2 (52 kDa) RelA (65 kDa), RelB (70 kDa) c-Rel (78 kDa)]	HNSCC cell line: UM-SCC-9, -11B, and -38	Constitutive and inducible NF-κB, and promoter activity	Overexpression of p65 in cell lines	Nil	(Duffey et al., 1999)
	Human HNSCC cell lines: UM-SCC-1, 9, 11A, 11B, 38	Constitutive DNA binding & promoter activity; Constituents p65/Rel A and p50; IL-8 induction: stronger role than AP-1	Nil	Nil	(Ondrey et al., 1999)
	Laryngeal SCC: Tumor and non-tumor laryngeal tissues	Nuclear positivity of p65	High levels of p65 in cytoplasm, moderate in nucleus	Nuclear p65 correlated with HPV16 E7 level	(Du et al., 2003)
	Oral tissue biopsies (n = 110). OSCC (n = 66) OPMD (n = 34), normal (n = 10); HPV positivity = 22%, HR-HPV16 = 18%	Constitutively active NF-κB; increased with disease severity; Major participants:p50, p65, p52, c-Rel, RelB, and Bcl-3	Upregulation of p50, p65 and c-Rel with increasing severity of lesion; immunoreactivity for p52, c-Rel and RelB in cancer tissues.	p50/p50 homodimerization common; Involvement of p65 only in NF-κB complex of HPV16-positive	(Mishra et al., 2006)
	HNSCC (n = 195); Control (n = 63; non cancer affected patients); Human HNSCC cell lines: Ho1N1, HSC2,and SKN3	SiRNA mediated downregulation of NF-κB activity; NF-κB nuclear staining: 55.6% HPV-negatives (above median-12.85), HPV-positive below median	Overexpression of RELA, NF-κB1	Differential gene signatures. Strong NF-κB activation in HPV-negative HNSCC	(Gaykalova et al., 2015)
	OC and OPSCC Tissue biopsies (Fresh, n = 116; and FFPE, n = 66)	Nil	Variable presence of p50 and p65 in HPV+ and HPV- tissues	p50: equal distribution among HPV-positive and HPV-negative tumors, p65: strong correlation with HPV-postive tumors	(Verma et al., 2017)
	Tongue tissue biopsies (n = 100) TSCC cell line: UPCI:SCC090 and AW13516	Increase d activity with severity of disease; p50 and c-Rel forming NF-κB	Differential expression of NF-κB proteins	Selective participation of p65 in NF-κB complex of HPV16-positive HNSCC	(Gupta et al., 2018)
**STAT3** (88 kDa)	HNSCC Cell line:YCU-N861, YCU-H891; HNSCC tissues (n = 6)	Constitutive activation of EGFR and STAT3; STAT3DN66 and STAT3DN99 abrogate STAT3 activity	Constitutive expression of STAT3	Nil	(Masuda et al., 2002)
	HNSCC tissues (n = 90)	High level of pSTAT3 in early stages (T1, T2), moderate in late stages (T3, T4); No STAT3 in normal samples	HNSCC - 82% with high or intermediate STAT3; No STAT3 in normal tssues	Nil	(Nagpal et al., 2002)
	HNSCC cell lines: HN6, HN12, HN13, HN30, HaCaT, HEK293T, HEK293FT cells; HNSCC FFPE tissues (n = 460)	pSTAT3 induction by IL-6 produced through active NF-κB signalling	STAT3 expression unaffected	Nil	(Squarize et al., 2006)
	HNSCC (n = 195); Control (n = 63; non cancer affected patients); Human HNSCC cell lines: Ho1N1, HSC2,and SKN3	Transcript profiling of STAT3 with NF-κB pathway signature target genes (IRF1, CEBPD, CCND1, ICAM1, JAG1, JAK3, and NOS3); reporter gene expression	Indirect evidence	Specific signature for HPV positive and negative; 49 HPV negative, while 1 HPV positive patients stained for nuclear STAT3 above median	(Gaykalova et al., 2015)
	OC and OPSCC Tissue biopsies (Fresh, n = 116; and FFPE, n = 66)	pSTAT3Y expression	Immunoblotting and Immunohistochemistry: inverse correlation between HPV positivity and STAT3/pSTAT3 expression.	HPV positive: low STAT3/pSTAT3; HPV negative: high STAT3/pSTAT3	(Verma et al., 2017)
**SOX2** (34 kDa)	OSCC frozen tumor tissue samples (n = 40)	SOX2 overexpression associated with increased activity	High expression of SOX2 and CyclinE1 in OSCC specimens	Nil	(Freier et al., 2010)
	HNSCC tumor tissues (n = 496)	–	SOX2 induced BCL-2 and enhanced chemo-resistance	SOX2 amplification in HPV-negative, but no amplification in HPV-positive	(Schrock et al., 2014)
	H&N tissue retrospectively collected (n = 94)	Nuclear positivity of SOX2	SOX2 expression detected in 95% of laryngeal dysplasia	Nil	(Granda-Diaz et al., 2019)
	OPSCC (n = 157)	Nil	SOX2 overexpression with poor prognosis	Poorer overall survival in SOX2-amplified HPV-negative cases (p = 0.036)	(Dogan et al., 2019)
	Tongue SCC cell line- CAL‐27	–	EGFR mediated stabilization and upregulation of SOX2 expression	Nil	(Lv et al., 2020)
**YY1** (44 kDa)	Laryngeal SCC Cell line - Hep-2; Control- HEK293T	Proliferation and migration with suppression of apoptosis as indicators of increased YY1 activity	YY1 upregulated in LSCC	Nil	(Qu et al., 2017)
	NPC cases (n = 40); Stage I replication (297 cases and 611 controls); Stage II replication (768 cases and 1526 controls)	YY1-mediated repression of TRIM26	Nil	Nil	(Lyu et al., 2018)
	OSCC tumor tissues (n-30); Cell lines- HEK293T, AW8507	YY1 mediated reporter gene activation by CARM1 mediated arginine methylation	YY1 overexpressed in oral cancer	Nil	(Behera et al., 2019)
**SP1** (80.6 kDa)	Normal oral mucosa (n-8), OSCC (n-10); OSCC cell lines: SCC-15, YD-15	SP-1 inhibitor downregulated expression of SP-1 and decrease tumor growth	SP-1 was overexpressed in oral tumors compared to normal	Nil	(Shin et al., 2013)
	NPC tumors (n-82), Metastatic malignant tumors (n-60), Nonmetastatic malignant tumors (n-22)	miR-24 overexpression lead to reduced SP1 activity and inhibited proliferation	Reduced SP1 expression contributed to the reduction in radio-resistance	Nil	(Kang et al., 2016)
	OSCC patients (n-55); Cell line- CAL-27 and SCC9	Upregulated SP-1 in OSCC	SP-1 is overexpressed in OSCC and could promote cell invasion and migration in OSCC	Nil	(Liu et al., 2019)
**OCT-1** (76.4 kDa)	HNSCC cell line: PCI-04A	–	Ionizing radiation induce OCT-1	Nil	(Meighan-Mantha et al., 1999)
**c/EBPβ** (76.4kDa)	HNSCC cell lines: UM-SCC-1, -9, -11A, -11B, and -38	Constitutive activation	–	Nil	(Ondrey et al., 1999)
	NPC and non-cancerous NPE (n-33); Cell line- HNE1 and 5–8F			Nil	(Wang et al., 2016)
**FOXA1** (49.1 kDa)/**FOXA2** (48.3 kDa)	HNSCC (n = 152)	Nil	FOXA1 expressed in basal cells of squamous epithelium, pre-invasive HNSCC lesions	Due to specificity of FOXA-1 positive site, it may have implication in HPV-mediated pathogenesis	(Karpathiou et al., 2017)
	NPC tissues (n-114), Non-cancer inflammatory NPE tissues (n-64)	FOXA1 regulated TGF-β-stimulated transcriptome	FOXA1 protein was decreased in NPC cells; loss of FOXA1 associated with lymph node metastasis and poor prognosis.	Nil	(Li et al., 2019)

AP-1, Activator Protein; DMBA, 7,12-dimethylbenz[a]anthracene; DN, Dominant Negative; EGFR, Epidermal Growth Factor Receptor; FFPE, Formalin-Fixed and Paraffin-Embedded; FOXA1, Forkhead Box A1; FOXA2, Forkhead Box A2; HPV, Human Papillomavirus; HNSCC, Head and Neck Squamous Cell Carcinoma; LSCC, Laryngeal Squamous Cell Carcinoma; NF-κB, Nuclear Factor-κB; NPC, Nasopharyngeal Carcinoma; NPE, Nasopharyngeal Epithelial; STAT3, Signal Transducer and Activator of Transcription – 3; SOX2, sex determining region Y (SRY)-box; SP1, Specificity Protein 1; TSCC, Tongue Squamous Cell Carcinoma; TPA, 12-O-tetradecanoylphorbol-13-acetate; OCT-1, Octamer Transcription Factor-1; OC- Oral Cancer; OSCC, Oral squamous Cell Carcinoma; OPSCC, Oropharyngeal Squamous Cell Carcinoma; OPMD, oral premalignant disease; VEGF, Vascular Endothelial Growth Factor; YY1, Yin Yang 1.

## Role of HPV-Related Transcription Factors in H&N Carcinogenesis

### AP-1

AP-1 was first discovered as TPA-inducible transcription factor that interacted selectively with sequences in the basal level enhancer of human metallothionein and showed binding to simian virus 40 enhancer region (Lee et al., 1987). Functional AP-1 is Lucine zipper family of transcription factors composed on homodimer of Jun family proteins (c-Jun, JunB and JunD) or its heterodimer with members of Fos family (c-Fos, FosB, Fra-1, and Fra-2). AP-1 is a redox sensor of the cell, which controls cell-proliferation, transformation, its members work as an oncogene [reviewed by (Angel and Karin, 1991; Shaulian and Karin, 2002)]. AP-1 proteins are primarily considered to be oncogenic but JunB, Fra-1 and Fra-2, have been shown to have tumor-suppressor activity (Eferl and Wagner, 2003). Therefore, oncogenic or tumor suppressive activity exhibited by distinct composition of AP-1 vary with cell context and the tumor genetic background. Due to the differential combination of the components, AP-1 is involved in a tissue-specific regulation of target genes. Considering the contrasting action of AP-1 complex, it has been referred as “double-edged sword” in tumorigenesis (Verde et al., 2007).

The first study that showed constitutively active AP-1 in human HNSCC tumors was incidentally meant to measure the activity of HIV LTR bearing AP-1 site (Zoumpourlis et al., 1994). An elevated AP-1 compared to adjacent normal tissues was reported in 7/7 tumor tissues studied. Later, AP-1 potentiated the action of NF-κB in basal and IL-1α-inducible expression of the pro-inflammatory cytokine, IL-8 in HNSCC cell lines (Ondrey et al., 1999; Wolf et al., 2001). AP-1 complex resulted in enhanced survival and proliferation of tumor cells (Wolf et al., 2001; Bancroft et al., 2002). EGFR-mediated signaling was found to contribute to expression and transactivation of AP-1, which participated more profoundly in the expression of angiogenic cytokine VEGF compared to IL-8 (Bancroft et al., 2001). Adverse effects of alcohol on oral epithelial cells are manifest by AP-1. Acetaldehyde, an ethanol metabolite, was found to activate c-Jun expression and dose-dependent activation of c-Jun containing AP-1 in human oral keratinocytes that were transformed by HPV oncoproteins (Timmons et al., 2002). Similarly, tobacco carcinogens independently promoted AP-1 activity, albeit through induction of *c-Fos* (Swenson et al., 2011). Metabolites of tobacco and alcohol together, promoted carcinogenesis with highly invasive AP-1 containing c-Jun and c-Fos in H&N region that block p53 (Schreiber et al., 1999). Our investigations on oral tissues revealed a weak/moderate level of most AP-1 members in oral mucosa except c-Fos, which was absent; or Fra-1 which showed high levels. AP-1 is seldom active in normal tissues (Mishra et al., 2010). While in precancer lesions, except Fra-1 expression of AP-1 proteins was upregulated and AP-1 activity was composed of JunD/JunD homodimers. Primary oral SCC (OSCC) showed heterodimers of c-Fos/JunD, which constituted the most prevalent AP-1 complex in cancer lesions (Mishra et al., 2010). In a murine metastasis model, HNSCC cell lines that showed lung metastatic potential were found to carry high differential expression of JunB, Fos, Fra-1 and JunD (Hyakusoku et al., 2016). Elevated expression of active phospho-c-Jun was typically reported in resistant tumors and it strongly correlated with bcl-2 overexpression (Alam et al., 2017). Although these studies emphasized the role of AP-1 in initiation, progression and chemoresistance of HNSCC, they did not address the heterogeneity within tumor types with respect to the HPV status and AP-1 components.

In a first landmark study carried out in mouse papilloma model that investigated the role c-Jun by expressing dominant negative c-Jun with HPV16 E7 showed that hyper-proliferative action of E7 was independent of c-Jun tumorigenic potential (Young et al., 2002). Similarly, c-Jun was found functional and got activated in HPV-transformed oral epithelial cells (Timmons et al., 2002). The observations showed that c-Jun, a common initiator in smoking-related AP-1 activation and HPV E7 action did not interfere with each other. Similarly, HPV and c-Jun expression did not show any correlation in premalignant and malignant oral tissues (Acay et al., 2008). Increased activation of AP-1 with increasing disease severity was seen in tongue SCC, which was accompanied with upregulation of downstream target genes: cyclin D1, c-myc, Bcl-xl, MMP-9, and EGFR (Gupta et al., 2015). With increasing disease severity, an increase in expression of all Jun and Fos family members was noted except Fra-1 which showed reciprocal kinetics. Selective participation of JunD and JunB was observed only in HPV16-positive tumors and cell lines, whereas c-Jun was absent in AP-1 complex of HPV-positive tumors. In a confirmatory immunohistochemical staining-based biomarker study on fresh and archival tissue, overexpression of JunD, and c-Fos was confirmed in SCC as compared to normal and an overexpression of JunB was seen only in HPV-positive tissues (Verma et al., 2017). These histological changes correlated well with p16 expression but showed no correlation to EGFR level of the tumor tissues. Overall these studies highlighted contrasting involvement of c-Jun in HPV-negative and JunB and JunD in HPV-positive tumors. Incidentally, c-Jun is known to downregulate p53 and p16 (Schreiber et al., 1999; Passegue and Wagner, 2000), whereas JunB was shown to upregulate the p16 expression (Passegue and Wagner, 2000). c-Jun also bypass PI3K signaling pathway and promote drug resistance by PI3K-independent AXL expression in HPV-negative tumors (Badarni et al., 2019). Further, HPV16 E6 was shown to contribute to AP-1 complex formation by inducing c-Fos after both ligand-dependent and independent EGFR activation (Liang et al., 2011). Independent of the HPV status, c-Jun has shown a poor prognosis in OSCC (Xu et al., 2017).

### NF-κB

Nuclear factor-κB was initially described as a nuclear factor required for the transcription of immunoglobulin κ light chain in B lymphocytes (Sen and Baltimore, 1986). NF-κB is counted among a few top pro-carcinogenic transcription factors. Presence of a Rel homology domain is the characteristic feature of all NF-κB members. Rel domain contains a nuclear localization sequence, sequence-specific DNA binding signature, assist dimerization, and interact with the inhibitory protein IκB.There are five proteins in the mammalian NF-κB family that are evolutionarily and structurally conserved and grouped in two classes, namely, Class-I [NF-κB1(p50) and NF-κB2(p52)] and Class-II [RelA(p65), RelB, and c-Rel) [reviewed by (Nabel and Verma, 1993)]. NF-κB/Rel proteins act both in homo- and in heterodimeric form. Most of the NF-κB dimers are activators of transcription; however, the p50/p50 and p52/p52 homodimers can repress the transcription of their downstream target genes (Zhong et al., 2002). NF-κB is an inducible transcription factor that controls the expression of several genes which regulate cell cycle (cyclin D1), cell survival (Bcl-2, Bcl-xL, cIAP), differentiation (p21^Cip/Waf1^), cell adhesion (VCAM, ECAM-1), growth factors (VEGF), angiogenesis (MMPs), and stemness (Bharti and Aggarwal, 2002; Rinkenbaugh and Baldwin, 2016).

Aberrant activation and dysregulation of NF-κB signalling is reported in many cancer types including colon, breast, pancreatic, leukemia, myeloma, cervical, and HNSCC (Bours et al., 1994; Dokter et al., 1995; Sovak et al., 1997; Wang et al., 1999; Bharti et al., 2003; Prusty et al., 2005; Gupta et al., 2018). NF-κB has a multifaceted role in HNSCC and is reviewed elsewhere (Monisha et al., 2017). Constitutively active NF-κB has been documented in HNSCC cell lines (Ondrey et al., 1999), and primary (Mishra et al., 2006; Bano et al., 2018; Gupta et al., 2018) and recurrent metastatic HNSCC (Li Z. et al., 2015). The expression and activity of NF-κB proteins changed as a function of severity of the oral lesions during oral cancer development (Mishra et al., 2006; Bano et al., 2018; Gupta et al., 2018). NF-κB DNA binding was constituted primarily by the homodimerization of p50 in HPV-negative HNSCC. Gene polymorphisms at *NFKB1* coding for p50 at locus rs28362491 have been found associated with susceptibility to HNSCC (Li and Zhang, 2019). Tobacco carcinogens were also found to induce NF-κB activity in HPV-transformed oral cavity cells (HOK 16B cells) (Rohrer et al., 2010).

Because of the overlapping role of NF-κB and AP-1 in inflammation and cancer, many studies investigated the role of these transcription factors together (Ondrey et al., 1999; Wolf et al., 2001; Chen et al., 2008) and their pharmacological targeting effectively attenuated expression of oncogenes HPV16 E6/E7 in HNSCC (Bancroft et al., 2002; Mishra et al., 2015). However, NF-κB showed a slightly skewed and stronger role in regulation of inflammatory mediators, whereas, AP-1 was found to control the angiogenic response. These observations fit well in the context of oral cavity. Local inflammation in periodontitis is associated with presence of soluble CD14 receptors in gingival crevicular fluid by oral epithelial cells (Nicu et al., 2009), which led to activation of NF-κB but not AP-1 (Feghali et al., 2011). This local inflammation and subsequent activation of NF-κB during an early HPV infection may promote LCR activity independent of AP-1 action. Gingival pockets have been referred as reservoirs of HPV and showed suitable environment including basal epithelium (Hormia et al., 2005). On the other hand, AP-1 gene network was reported to enhance epithelial repair in nasal polyposis by oral steroids (Li et al., 2009).

Dissection of NF-κB complex in HNSCC revealed a strong NF-κB activity composed of p50:p50 homodimer in HPV-negative lesions, whereas HPV16-positive lesions showed comparatively lower activity with additional participation of p65 and c-Rel (Mishra et al., 2006). Earlier a moderate intra-nuclear staining compared to high cytoplasmic p65 was noted in HPV16 E7 expressing laryngeal cancer cells earlier (Du et al., 2003). Incidentally, involvement of p65 also resulted in cisplatin-resistance observed in HNSCC (Li Z. et al., 2015). NF-κB activation was negatively influenced by HPV16 E7 by blocking upstream signaling (Spitkovsky et al., 2002) or induced by HPV16 E6 through its PDZ-binding motif (James et al., 2006). A detailed differential gene expression profile analysis revealed a STAT3 and NF-κB target gene signatures could effectively distinguish HPV-positive from HPV-negative HNSCC (Gaykalova et al., 2015). Later, we discovered that direct evaluation of NF-κB active members in association with active STAT3 in immunohistochemical staining can also discriminate HPV-positive from HPV-negative HNSCC. NF-κBp50 was associated with active STAT3 in HPV-negative HNSCC, whereas NF-κBp65 expression and lack of STAT3 was associated with HPV-positive HNSCC (Verma et al., 2017). Co-activated NF-κB and STAT3, often observed in HPV-negative tumors, strongly promoted cell survival through upregulation of BAX/BCL-XL expression in HNSCC (Lee et al., 2008).

Active NF-κB in HPV-positive lesions was found associated with better physical performance of HNSCC patients (Xiao et al., 2018). Coactivation of both classic and alternate NF-κB pathways collectively translates into inflammatory transcriptome in HNSCC (Yang et al., 2019). Recent reports suggest role of TRAF3, often found compromised in HPV-positive HNSCC (Hajek et al., 2017) in preventing nuclear localization of alternate NF-κB complex with p52/RelB constituents in HPV-negative HNSCC (Zhang et al., 2018). Thus, the presence of NF-κB is essential in all types of HNSCC; however, the subtle changes in their composition, activity, expression profile, and target genes correlate with HPV positivity and may also contribute to the prognosis.

### STAT3

STAT3 belongs to the family of latent cytoplasmic proteins that are directly activated by tyrosine kinases (Schindler et al., 1992; Shuai et al., 1992) and regulate expression of key genes involved in development, inflammation and wound healing [reviewed by (Yu and Jove, 2004; Leeman et al., 2006; Lai and Johnson, 2010; Johnson et al., 2018)]. STAT3 particularly, is an established oncogene and its aberrant expression and activation is known to promote epithelial carcinogenesis (Zhong et al., 1994; Kim et al., 2007; D’amico et al., 2018). STAT3 has been associated with several types of epithelial and haematological malignancies. Constitutive activation of STAT3 is also a central feature of HNSCC (Song and Grandis, 2000). STAT3 modulates the expression of multitude of genes that are involved in cell proliferation, differentiation, apoptosis, cell cycle regulation, angiogenesis, EMT and immune invasion during development of HNSCC [reviewed by (Lai and Johnson, 2010; Masuda et al., 2010; Johnson et al., 2018)]. STAT3 activation occurs early during HNSCC carcinogenesis due to the autocrine activation of TGF/EGFR signalling (Grandis et al., 1998; Grandis et al., 2000).

Immunohistochemical analysis and protein micro-arrays studies in primary HNSCC tissue specimens have demonstrated constitutive activation of STAT3 (Nagpal et al., 2002; Shah et al., 2006; Weber et al., 2007). An overexpression with constitutively active STAT3 construct in HNSCC cells showed Cyclin D1 overexpression, increased proliferation *in vitro* and higher rates of *in vivo* tumor growth in mice (Kijima et al., 2002; Masuda et al., 2002). Inhibition of STAT3 using a dominant negative STAT3 construct, antisense oligonucleotides, a transcription factor decoy or siRNA resulted in decreased saturation density, increased serum dependence, growth inhibition and induction of apoptosis (Grandis et al., 1998; Grandis et al., 2000; Masuda et al., 2002; Leong et al., 2003; Gao et al., 2005; Gao et al., 2006). Further, siRNA administration against STAT3 in the xenograft model demonstrated a decrease in tumor volume and induction of apoptosis (Leong et al., 2003). In contrast, suppression of STAT3 in HNSCC cells promoted secretion of both pro-inflammatory chemokines and cytokines (Albesiano et al., 2010) thus suggestive of immunosuppressive function of STAT3 in HNSCC. Activation of STAT3 upregulated the immune checkpoints PD-1/PD-L1 expression, whereas downregulation of pSTAT3 in the absence of EGFR and PTEN decreased the PD-1/PD-L1 expression (Bu et al., 2017). A differential expression of STAT3 in subtypes of oral lichen planus have recently been noted to contribute to disease progression (Du et al., 2018).

Except in some studies (Pectasides et al., 2010; Mazibrada et al., 2014), presence of active nuclear STAT3 in HNSCC was invariably associated with poor prognosis (Masuda et al., 2010; Macha et al., 2011; Jinno et al., 2015; Gao et al., 2016; Lesinski et al., 2019). Emerging research on the functional regulatory role of STAT3 revealed its strong correlation with particularly HPV-negative HNSCC making it a compelling target particularly in HPV-negative HNSCC (Gaykalova et al., 2015). HPV positive HNSCC, however, showed a strong negative correlation with active STAT3 in HNSCC (Verma et al., 2017), which correlated well with the differential transcript profile of STAT3 target genes (Gaykalova et al., 2015). Elevated STAT3 and upstream signalling in HPV-positive HNSCC have been reported in some studies (Mazibrada et al., 2014; Chuerduangphui et al., 2016), though the reason behind such contrasting observations is unclear. In sharp contrast to HNSCC, STAT3 in cervical cancer was found aberrantly expressed and constitutively activated in HPV-induced cervical carcinogenesis (Shukla et al., 2010; Shukla et al., 2019) and the level of active STAT3 was found strongly correlated with the physical state of HPV genome in cervical precancer and cancer tissues (Shukla et al., 2019). Therefore, the involvement of STAT3 in HPV-positive epithelial malignancies is a tissue context dependent; however, its influence on pathological manifestation of HPV genome cannot be overlooked in HNSCC. It can be hypothesized that high STAT3 might upregulate HPV replication involved in an early phase of life cycle through HPV E6-driven NF-κB (Morgan et al., 2018; Morgan and Macdonald, 2019). However, such assumption needs to be tested rigorously in experimental systems.

### SOX2

*SOX2* gene was discovered in 1994 (Stevanovic et al., 1994). SOX2 transcription factor is a member of the SoxB1 group (Kijima et al., 2002; Leong et al., 2003). The protein family has a highly conserved DNA binding HMG (High mobility group) box consisting of about 80 amino acids. Sox proteins function majorly in embryonic development and cell fate determination. SOX2 is involved in stem cell maintenance. Amplification of SOX2 is common in different cancer types, which adversely effects cancer cell physiology *via* altering stem cell signaling pathways (Bass et al., 2009; Wuebben and Rizzino, 2017). SOX2 is activated by recurrent 3q26.33 amplifications in squamous cell carcinoma (Hussenet et al., 2010).

In HNSCC expression of SOX2 is correlated with poor prognosis (Leeman et al., 2006; Weber et al., 2007). SOX2 upregulates Cyclin B1 and promotes cell proliferation and leads to HNSCC cell dedifferentiation (Lee et al., 2014). In oral carcinogenesis, SOX2 is activated *via* gain of gene copy number (Freier et al., 2010). In laryngeal cancer, SOX2 promotes migration and invasion by inducing MMP-2 *via* the PI3K/Akt/mTOR pathway (Yang et al., 2014). SOX2 was found during early tumorigenesis of laryngeal cancer and its expression could be used as independent predictor of laryngeal cancer risk in patients with precancerous lesions (Granda-Diaz et al., 2019). On the contrary, EGFR in HNSCC enhances the stemness and progression of oral cancer through inhibition of SOX2 degradation (Lv et al., 2020). SOX2 is a marker for cancer stem cell in head and neck cancer (Dong et al., 2014), and is a prognostic marker with unfavorable outcome (Germain and Frank, 2007). SOX2 induced expression of the antiapoptotic protein BCL-2 and enhanced resistance to apoptosis-inducing chemotherapy agents including cisplatin, indicating SOX2 as a mediator of chemotherapy resistance in human HNSCC (Schrock et al., 2014). Further, HPV-positive HNSCC lacked SOX2 amplification and lower SOX2 protein expression as compared to HPV-negative tumors. SOX2 overexpression was associated with poor survival and negative HPV status of OPSCC (Dogan et al., 2019). Three putative sites of SOX2 were present exclusively in the enhancer region of HPV16-LCR and overexpression of this transcription factor led to repression of transcriptional activity of HPV16-LCR by decreasing the expression of E6 and E7 endogenous HPV oncogenes (Martinez-Ramirez et al., 2017). Notably, SOX2 is expressed in normal stem cells of gingiva (Soanca et al., 2018), was detectable in oral leukoplakia (Luiz et al., 2018) and have been implicated in oral carcinogenesis (Qiao et al., 2014).

### Glucocorticoid Receptor (GR)/Progesterone Receptor (PR)

Glucocorticoid receptor is a hormone-inducible transcription factor (Ostlund Farrants et al., 1997). Glucocorticoids are a class of corticosteroids and act as ligand for glucocorticoid receptor (GR) (86 kDa) is an evolutionally conserved nuclear receptor superfamily protein and mediates diverse actions of glucocorticoid hormones by acting as a ligand-dependent transcription factor. In absence of ligand, GR reside primarily in cytoplasm of cell as a part of large multiprotein complex (receptor polypeptide and two molecules of hsp90 and other proteins), after interaction with ligand it dissociates from hsp and translocates to nucleus. In nucleus, GR binds to glucocorticoid receptor element (GRE) in promoter region of target genes (Tomoshige, 2017). In regulating stress response, a negative cross talk between AP-1 and GR has been reported which states that AP-1 increased level of circulating glucocorticoids, which reciprocally downregulated AP-1 transcriptional activity (Karin and Chang, 2001). A complex of AP-1 and GR function together as a composite site in HPV16 expression (Mittal et al., 1994). In nasal polyposis, steroids synergize with AP-1 to enhance epithelial repair through upregulation of AP-1 gene network (Li et al., 2009).

Glucocorticoids inhibit NF-κB activation as active GR binds to p65 subunit and prevents activation of inflammatory genes (Didonato et al., 1996; Barnes and Karin, 1997). GR interacts with NF-1, this interaction modulates activity of promoter in mouse mammary tumor virus (MMTV) (Hartig and Cato, 1994). GR inhibits p53 functions by forming a complex that mutually represses each other’s transcriptional activity (Sengupta et al., 2000). In HPV16 E6 and E7 oncogenes expression increased by steroid hormones and degraded p53 gene product and led to carcinogenesis (Moodley et al., 2003). GR has been reported to be a transcription factor associated with the regulation of HPV gene expression. HPV16 LCR contains enhancer element for GR (Moodley et al., 2003). Glucocorticoid hormone exerts regulatory action on HPV16 gene expression during growth and differentiation of cervical cancer (Khare et al., 1997). In prostate cancer GR increases radioresistance and triggers androgen hormone independence (Chen et al., 2019). However, there is no study so far on GR in relation to HNSCC.

Progesterone receptor (PR) (99 kDa) is a master regulator in female reproductive tissues that controls developmental processes, proliferation and differentiation during the reproductive cycle and pregnancy, however, its expression in low in males. GR and PR transcription factors bind to same consensus sequence (Jacobsen and Horwitz, 2012; Grimm et al., 2016). PR after engaging with the Progesterone dimmerizes and translocates to the nucleus. Hormone in oral contraceptive pills have been reported to enhance tumorigenesis in cervical region (De Villiers, 2003). Increased PR expression has been associated with the proliferation of normal cervical squamous epithelium, which is probably induced by HPV infection, however this is typical in neoplastic cervical squamous epithelium (Konishi et al., 1991; Monsonego et al., 1991). In contrast, high PR expression was associated with poor disease-specific and locoregional recurrence-free survival in OSCC in HPV-negative tumors (Mohamed et al., 2018).

### Yin Yang 1 (YY1)

Yin Yang 1 is ubiquitously expressed, evolutionarily conserved zinc finger protein that belongs to the GLI-Krüppel gene family (Shi et al., 1991). It plays a critical regulatory role in expression of various gene sets and its function in cancerous environment is subtle (Thiaville and Kim, 2011). YY1 acts as a transcriptional repressor (Galvin and Shi, 1997), and plays oncogenic and proliferative role in carcinogenesis (Zhang et al., 2011). Oncogenic role of YY1 in different cancers have been shown (Seligson et al., 2005; Chinnappan et al., 2009; Lee et al., 2012; Wan et al., 2012; Zhang et al., 2013; Zhang J. J. et al., 2014; Liu D. et al., 2018). YY1 binds with transcriptional coactivator CREB-binding protein and represses AP-1 activity. Highly conserved AP-1 site was identified as a target of YY1 mediated repression in HPV LCR (O’connor et al., 1996). The binding of YY1 to its cognate site interferes with the formation of HPV16 transcription initiation complex at the P97 promoter for the E6/E7 genes (O’connor et al., 1996). Sequence analysis of the HPV16 LCR from *in vitro* HNSCC model expressing high level of E6 gene expression revealed a deletion of 163 bp that included two binding sites of YY1 known to negatively regulate HPV16 E6 and E7 expression (Ferris et al., 2005). YY1 promotes migration, proliferation and suppression of apoptosis in laryngeal cancer *via* directly inhibiting MYCT1, which can be used as target to prevent the progression of laryngeal cancer (Qu et al., 2017). YY1 attenuates viral early gene expression *via* CTCF-YY1-dependent looping in the HPV-infected undifferentiated cells. However, during cellular differentiation, YY1 protein expression is dramatically reduced and viral oncogenes E6 and E7 are upregulated, suggesting that CTCF (chromatin-organizing CCCTC-binding factor)-YY1 loop could be disrupted in HPV-mediated carcinogenesis (Pentland et al., 2018). Downregulation of YY1 has been correlated with low immunogenic role in nasopharyngeal carcinoma (Lyu et al., 2018). YY1 in association with CARM1 promotes oral cancer carcinogenesis (Behera et al., 2019). YY1 modulates inflammatory cytokines indirectly in gingival epithelial cells (Nakayama et al., 2019). Therefore, presence of high level of YY1 in pre-neoplastic tissues prevents transcriptional activation of HPV and takes the tumor to a fate resembling HPV-negative tumors.

### Nuclear Factor–1 (NF-1)

NF-1 is a family of ubiquitous nuclear transcription factors with subtypes NF-1A (55 kDa), NF-1B(47 kDa), NF-1C(55.6 kDa), and NF-1X(48.8 kDa), which specifically bind as dimers to the palindromic consensus DNA sequence. NF-1 is also known as CAAT box-binding transcription factor (CTF). Oxidative inactivation plays an important role in the DNA-protein interaction, considerably reducing the affinity of the transcription factor NF-1 for DNA (Bandyopadhyay and Gronostajski, 1994). NF-1 family CTF/NF-1 transcription factors act as potent genetic insulators for integrating gene transfer vectors (Gaussin et al., 2012). The strict correlation between the activation or lack of activation of the HPV16 enhancer and cell-specific subsets of NF-1 proteins argues for the pivotal role of NF-1 binding sites in the epithelial cell-specific function of the viral enhancer (Apt et al., 1993). NF-1 and p53 play an important role in glioma initiation and progression (Gonzalez et al., 2018). In HPV16 genome NF-1 binding sites were found methylated in high grade disease, in cervical cancer methylation percentages of E6 and E7 CpG were elevated (Kottaridi et al., 2019). In HPV11, mutation in NF-1 binding site reduces promoter activity in differentiating cells (Zhao et al., 1999). Despite a strong redox regulated transcription factor related to HPV, status and role of NF-1 in HNSCC has not been evaluated till date.

### Specificity Protein 1 (SP1)

Specificity Protein 1 (SP1) belongs to SP/KLF transcription factor family, which represents the major group of zinc-finger DNA binding proteins. It was first identified as a promoter‐specific binding factor that is essential for transcription of the SV40 major Immediate Early (IE) gene and shown to interact with GC and GT oligonucleotide sequences that are typically found in diverse viral and cellular gene promoter (Dynan and Tjian, 1983; Safe and Kim, 2004). It is estimated that at least 12,000 SP1 binding sites are present within human genome, and most studies support the idea that SP1 not only maintains basal transcription, but also contributes to the regulation, i.e., induction and inhibition, of transcription of a large number of cellular genes (Cawley et al., 2004; Oleaga et al., 2012).

SP1 is overexpressed in cancer cells and upregulates genes that enhance proliferation, invasion, metastasis stemness and chemoresistance. It is reported to be over‐expressed in a number of cancers, including breast (Oleaga et al., 2012), gastric (Yao et al., 2004), pancreatic (Jiang et al., 2008), lung (Hsu et al., 2012), glioma (Guan et al., 2012) and thyroid cancer (Chiefari et al., 2002). SP1 regulates the metastasis in LSCC, promotes cell migration and invasion in OSCC by upregulating Annexin A2 transcription, and downregulation of SP1 inhibits the growth of oral cancer cell SCC-15, YD-15 (Cai et al., 2012; Shin et al., 2013; Liu et al., 2019). Promoters of HPV are known to have the binding site of SP1 transcription factor which activates the transcription of HPV16 genes and regulate the replication of HPV18 (Tan et al., 1992; Demeret et al., 1995; Apt et al., 1996).

Overexpression of SP1 has been observed in HNSCC as compared to normal oral mucosa, and its inhibition downregulates growth in oral cancer cell lines (Shin et al., 2013). SP1 serves as a therapeutic target against oral cancer (Shin et al., 2012; Yu et al., 2013; Chae et al., 2014; Sun et al., 2016; Zhang et al., 2019; Zhao et al., 2019; Gao et al., 2020; Liu et al., 2020). SP1 is a functional target of miR-24. Nasopharyngeal carcinoma showed low level of miR-24. When miR24 was ectopically expressed in these cells, radiosensitivity was increased with a decline in level of SP1 (Kang et al., 2016). Involvement of SP1 with PI3K pathway was reported in EFGR expression by activin A in HNSCC (Tsai et al., 2019). Cell migration and invasion of OSCC was promoted by SP1 through Annexin A2. Annexin A2 is an oncogene, which has three putative binding sites of SP1 at its promoter region (Liu et al., 2019). Despite a critical transcription factor, there are only limited studies on this transcription factor with no HPV-specific information in HNSCC.

### Transcriptional Enhancer Factor-1 (TEF-1)

TEF-1 (47.9 kDa) is also known as TEAD1- TEA domain family member 1. It was the first known transcription factor of TEAD family (Xiao et al., 1991). TEF-1 is a part of Hippo signaling pathway which is involved in organ size control and act as a tumor suppressor by restricting proliferation and promoting apoptosis. TEF-1 functions majorly in development of skeletal and cardiac muscle (Chen et al., 1994; Anbanandam et al., 2006). On HPV16 p97 promoter region, binding of TEF-1 is necessary for its activity, both TEF-1 and its co-activator are essential for transcription of HPV16 and are found active in human keratinocytes (Ishiji et al., 1992). A TATA-less promoter containing binding sites for ubiquitous transcription factors mediates cell type-specific regulation of the gene for TEF-1 (Boam et al., 1995). Even though TEF-1 is essential for HPV transcription, relation of TEF-1 in HNSCC has not been explored as yet.

### OCT-1

Oct1 (POU2F1), stands for Octamer transcription factor-1 and is a OCT/POU family member, which was identified as a protein associated with regulatory DNA binding sites in animal viruses [adenovirus (Pruijn et al., 1986), SV-40 (Pruijn et al., 1986), and herpes simplex virus-1 (Gerster and Roeder, 1988)]. It binds to octamer motif (5’-ATTTGCAT-3’) and activates the promoters of variety of genes and of small nuclear RNAs (snRNA). OCT-1 regulates the genes associated with proliferation and immune modulation. Its recently identified targets are associated with oxidative and cytotoxic stress resistance, metabolic regulation, and stem cell function (Vazquez-Arreguin and Tantin, 2016). OCT-1 regulates gene expression *via* nuclear organization, controls the transcriptional regulation and affects tumor development (Malhas et al., 2009). OCT-1 mediates epithelial-mesenchymal transition in colorectal cancer and is associated with tumor progression and poor patient survival (Li Y. et al., 2015). Similar to some of basal transcription factors stated above, role of this transcription factor in HNSCC and HPV infection remains undefined.

### CCAAT Enhancer Binding Protein Beta (c/EBPβ)

c/EBPβ/NF-IL6 belongs to basic leucine zipper family domain. It can also form heterodimers with the related proteins c/EBPα, c/EBPδ, and c/EBPγ. c/EBPβ transcription factor is involved in cell proliferation and tumorigenesis (Ondrey et al., 1999). c/EBPβ expression is stimulated with IL-6, glucogon, IL-1, lipopolysaccharide (LPS), and glucocorticoid, which suggests its role in the mediation of the inflammatory response (Akira et al., 1990; Alam et al., 1992; An et al., 1996; Matsuno et al., 1996). c/EBPβ is constitutively active in HNSCC cell lines (Ondrey et al., 1999). c/EBPβ act as repressor for HPV11 in keratinocytes as levels of both HPV11 transcripts and HPV DNA increased after treatment with oligomers containing the c/EBPβ DNA binding motif that neutralized endogenous c/EBPβ (Wang et al., 1996). A novel c/EBPβ-YY1 complex that binds the switch region and contributes to cell-type-specific HPV-18 LCR activity  (Bauknecht et al., 1996). c/EBPβ is a repressor of E6/E7 promoter and its binding site overlaps with AP-1 in HPV16 LCR  (Liu et al., 2002). HPVE2 interacts with c/EBP factors, and enhances keratinocyte differentiation in HPV-induced lesions (Hadaschik et al., 2003). The role of c/EBPβ in HNSCC has not been explored yet.

### Forkhead Box A1 (FOXA1)/Forkhead Box A2 (FOXA2)

FOXA1 (Forkhead Box A1) and FOXA2 (48.3 kDa) are members of FOX (forkhead box) family. FOXA1 is also known as Heaptocyte Nuclear Factor 3 (HNF3α) and FOXA2 as (HNF3β) (Xanthopoulos et al., 1991; Bingle and Gowan, 1996). Forkhead proteins play an essential role in Estrogen receptor (ER) binding and ER-mediated gene transcription (Carroll et al., 2005). An oncogenic role of HNF3α (FOXA1) was observed in the progression and development of lung and esophageal adenocarcinoma (Yu and Jove, 2004). FOXA1 served as an important mediator for transcriptional activation of *p16* gene induced by replicative and oncogene-induced senescence (Schindler et al., 1992). Role of FOXA1 has been elaborated in many cancers, prostate cancer cell proliferation and cancer progression was mediated through FOXA1 by targeting the tumor suppressor gene IGFBP-3 (Song and Grandis, 2000). Expression of FOXA1 can be used as a biomarker to differentiate the breast carcinoma from other carcinomas (Heinrich et al., 1998). Growth in lung adenocarcinoma was regulated by FOXA1 and FOXA2 activity (Grandis et al., 2000). FOXA1 is expressed in basal cells of squamous epithelium in dysplastic and invasive lesions of cervical and head and neck carcinomas (Yue and Turkson, 2009). FOXA1 is able to bind with HPV genome and, therefore, acts as a potential positive regulator (Shuai et al., 1992). FOXA1 is found in pre-cancerous lesions of head and neck origin supporting its role in HPV pathogenesis (Cripe et al., 1987).

Similarly, FOXA2 also has oncogenic potential and its role has been elucidated in many cancers. In uterine cancer, FOXA2 is frequently mutated and act as a pathogenic driver gene in the etiology of this cancer (Le Gallo et al., 2018). In pancreatic cancer, FOXA2 controls the *cis*-regulatory networks in a differentiation grade-specific manner (Milan et al., 2019). FOXA2 is overexpressed in ovarian cancer stem cells and regulates its autophagy activity (Peng et al., 2017). FOXA2 inhibited EMT in breast cancer cells by regulating the transcription of EMT-related genes such as E-cadherin and ZEB2 (Zhang et al., 2015). FOXA2 has a tumor suppressor function through inhibition of pancreatic cancer cell growth, migration, invasion, and colony formation (Vorvis et al., 2016). The role of FOXA2 in HNSCC has not been elucidated so far.

## Discussion

Overall, the evidence presented above demonstrates that HPV positive and negative HNSCC are clearly two distinct entities that notably differ in their clinico-pathological and molecular characteristics. The striking difference in disease progression of two lesion types may be associated with a differential expression and activity profile of key transcription factors responsible for transcriptional activity of HPV oncogenes. Apart from regulation of oncogenic HPVs, the pro-carcinogenic role of many of these transcription factors is well-established. Any change/absence of this HPV-permissive profile in host cell milieu could be the rationale behind clearance of HPV infection or absence of transcriptionally-active HPV leading to a different pathologic manifestation and treatment response of the disease that resemble HPV-negative HNSCC. Therefore, such tumors even though are positive for HPV DNA, fail to show better prognosis.

**Figure 6:**
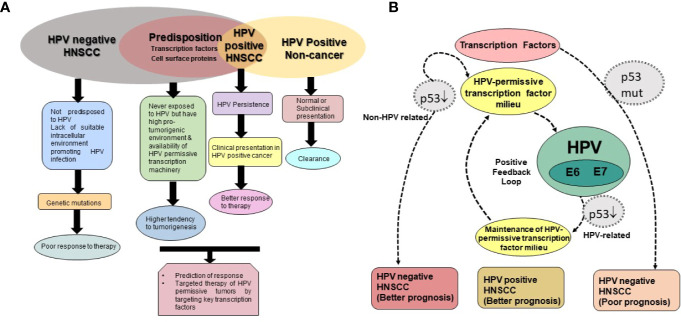
Transcription factor-based classification of HPV positive and HPV negative HNSCC. **(A)** Schematic representation of molecular sub-types of HNSCC. HPV negative HNSCC collectively represent tumors with availability of HPV-permissive transcription factors that are never exposed to HPV infection in the lifetime and HPV negative tumors, which are driven by genetic mutations in p53 or other such genes. The later represent tumors with poorer prognosis. Further, not all individuals that encounter HPV in H&N region develop cancer. HPV positivity is reported in oral rinse of normal individual. HPV-permissive transcription factor cocktail if present is expected to direct treatment outcome similar to the tumor-tissue with HPV-driven transcription profile. In the absence of HPV infection, such cases go unnoticed and clubbed with HPV negative HNSCC and could be effectively treated with less aggressive treatment regimen and likely to have a better treatment outcome. **(B)** Expression of viral oncogenes under the influence of HPV-permissive transcription factor milieu lead to functional inactivation of p53 and feeds to a positive feedback look that maintains the HPV-permissive transcription factor milieu. On the other hand, HPV negative tumors, particularly with mutations in p53, in consort with other procarcinogenic transcription factors like STAT3 drive tumors that show poorer response to anti-cancer therapies.

Despite HPV positive HNSCC represent as a separate class, all tissues of H&N region or all individuals affected by HNSCC may not have the opportunity of oral HPV exposure during initiation or progression of the disease. However, there may be an underlying predisposition of the tumor tissues in terms of availability HPV-permissive transcription factor cocktail and downstream transcriptome (**Figure 6**). In the absence of HPV infection, such cases go unnoticed and clubbed with HPV-negative HNSCC and could be effectively treated with less aggressive treatment regimen. This special class of HPV-positive HNSCC is expected to have a better treatment outcome. However, this hypothesis remains to be tested in a clinical set-up.

Present review and the evidence presented herein strongly support towards a need to collectively monitor activity and expression of these HPV-permissive transcription factors. However, required molecular diagnostic test to address these parameters is currently non-existent. In the absence of a direct test(s), the activity of these transcription factors can be assessed by evaluating each transcription factor separately (Mishra et al., 2006; Hussain et al., 2009; Shukla et al., 2010; Gupta et al., 2015; Mishra et al., 2015; Verma et al., 2017; Gupta et al., 2018), or by evaluating the transcripts of their respective downstream target genes by next generation sequencing, or similar platforms as described (Gaykalova et al., 2015). Apart from serving as prognostic tools, these potent procarcinogenic transcription factors may serve as molecular targets for pharmacological interventions in both HPV-positive and HPV-negative HNSCC.

## Author Contributions

NA participated in study writing and manuscript preparation. JY, KT, RB, AC, and TT contributed in manuscript preparation. MK assisted in manuscript preparation. ACB conceived the presented idea and designed the manuscript, critically reviewed, and drafted and communicated the final manuscript. All authors contributed to the article and approved the submitted version.

## Funding

The study was supported by research funds to ACB from the Department of Biotechnology, Government of India (DBT:6242-P34/RGCB/PMD/DBT/ALCB/2015) and Senior Research Fellowship to KT, AB and MJ from Indian Council of Medical Research (5/13/38/2014 NCDIII-Eoffice73143), (2017-2834/CMB/BMS) and (3/2/2/278/2014-NCD III); Junior Research Fellowship to NA (09/045(1622)/2019-EMR-I), JY (09/045(1629)/2019-EMR-I), RB (09/045(1672)/2019-EMR-I) and US (09/045(1584)/2018-EMR-I) by Council of Scientific and Industrial Research (CSIR); Senior Research Fellowship to TS (2061430699 22/06/2014(i)EU-V), and Junior Research Fellowship to AC [573(CSIR-UGC NET JUNE 2017)] by University Grants Commission (UGC); Junior Research Fellowship to TT by Department of Science and Technology, India (EMR/2017/004018/BBM); and The Teachers Associateship for Research Excellence (TARE) from the Science and Engineering Research Board (SERB), India to MKK (TAR/2018/001054).

## Conflict of Interest

The authors declare that the research was conducted in the absence of any commercial or financial relationships that could be construed as a potential conflict of interest.

## Abbreviations

AP-1, activator protein-1; APC, annual percentage change; CT, chemotherapy; DFS, disease free survival; ECOG, Eastern Cooperative Oncology Group; EGFR, epidermal growth factor receptor; FFPE, formalin-fixed, paraffin-embedded; HPV human papillomavirus; HR, hazard ratio; H&N, head and Neck; HNSCC, head and neck squamous cell carcinoma; NF-κB, nuclear factor-kappa B; OPSCC or OPC, oropharyngeal squamous cell carcinoma; OR-odds ratio; OS, overall survival; OSCC, oral squamous cell carcinoma; PCR, polymerase chain reaction; PFS, progression-free survival; PIK3CA, phosphatidylinositol-4,5-bisphosphate 3-kinase catalytic subunit alpha; R/M, recurrent and/or metastatic; SCC, squamous cell carcinoma; SEER, Surveillance, Epidemiology, and End Results program registries; STAT3-signal transducer and activator of transcription 3; TRAF3, TNF receptor associated factor 3; UADT, upper aero digestive tract; WDSCC, well differentiated squamous cell carcinoma.
